# Chloropyridinyl Esters of Nonsteroidal Anti-Inflammatory Agents and Related Derivatives as Potent SARS-CoV-2 3CL Protease Inhibitors

**DOI:** 10.3390/molecules26195782

**Published:** 2021-09-24

**Authors:** Arun K. Ghosh, Dana Shahabi, Monika Yadav, Satish Kovela, Brandon J. Anson, Emma K. Lendy, Connie Bonham, Devika Sirohi, Carlos A. Brito-Sierra, Shin-ichiro Hattori, Richard Kuhn, Hiroaki Mitsuya, Andrew D. Mesecar

**Affiliations:** 1 Department of Chemistry; Department of Medicinal Chemistry, Purdue University, West Lafayette, IN 47907, USA; dshahabi@purdue.edu (D.S.); yadav21@purdue.edu (M.Y.); satish.kovela@gmail.com (S.K.); 2 Department of Biological Sciences, Purdue University, West Lafayette, IN 47907, USA; banson@purdue.edu (B.J.A.); dsirohi@purdue.edu (D.S.); cbritosi@purdue.edu (C.A.B.-S.); kuhnr@purdue.edu (R.K.); amesecar@purdue.edu (A.D.M.); 3 Department of Biochemistry, Purdue University, West Lafayette, IN 47907, USA; elendy@purdue.edu (E.K.L.); 4 Bindley Bioscience Center, Purdue University, West Lafayette, IN 47907, USA; bonhamc@purdue.edu; 5 Purdue Institute of Inflammation, Immunology and Infectious Disease, Purdue University, West Lafayette, IN 47907, USA; 6 Departments of Hematology and Infectious Diseases, Kumamoto University School of Medicine, Kumamoto 860-8556, Japan; shattori@ri.ncgm.go.jp (S.-i.H.); hmitsuya@hosp.ncgm.go.jp (H.M.); 7 Department of Refractory Viral Infections, National Center for Global Health and Medicine Research Institute, Tokyo 162-8655, Japan; 8 Experimental Retrovirology Section, HIV and AIDS Malignancy Branch, National Cancer Institute, Bethesda, MD 20892, USA

**Keywords:** indomethacin derivative, antiviral activity, COVID-19, 3CLpro inhibitors, covalent inhibitors, ibuprofen derivative, SARS-CoV-2, salicylic acid derivative

## Abstract

We report the design and synthesis of a series of new 5-chloropyridinyl esters of salicylic acid, ibuprofen, indomethacin, and related aromatic carboxylic acids for evaluation against SARS-CoV-2 3CL protease enzyme. These ester derivatives were synthesized using EDC in the presence of DMAP to provide various esters in good to excellent yields. Compounds are stable and purified by silica gel chromatography and characterized using ^1^H-NMR, ^13^C-NMR, and mass spectral analysis. These synthetic derivatives were evaluated in our in vitro SARS-CoV-2 3CLpro inhibition assay using authentic SARS-CoV-2 3CLpro enzyme. Compounds were also evaluated in our in vitro antiviral assay using quantitative VeroE_6_ cell-based assay with RNAqPCR. A number of compounds exhibited potent SARS-CoV-2 3CLpro inhibitory activity and antiviral activity. Compound **9a** was the most potent inhibitor, with an enzyme IC_50_ value of 160 nM. Compound **13b** exhibited an enzyme IC_50_ value of 4.9 µM. However, it exhibited a potent antiviral EC_50_ value of 24 µM in VeroE6 cells. Remdesivir, an RdRp inhibitor, exhibited an antiviral EC_50_ value of 2.4 µM in the same assay. We assessed the mode of inhibition using mass spectral analysis which suggested the formation of a covalent bond with the enzyme. To obtain molecular insight, we have created a model of compound **9a** bound to SARS-CoV-2 3CLpro in the active site.

## 1. Introduction

Novel Coronavirus disease 2019 (COVID-19) caused by severe acute respiratory syndrome coronavirus 2 (SARS-CoV-2) is responsible for the ongoing COVID-19 pandemic [1,2]. The first cases of the disease were reported in Wuhan, China and then rapidly spread worldwide, overwhelming health care systems, disrupting economies, and leading to extensive loss of human lives [3,4,5]. At present, there is no specific efficacious treatment for COVID-19, except remdesivir, which shows only modest benefits to patients with COVID-19 infection [6,7]. Current vaccination efforts are showing benefits, but the hope that COVID-19 will be under control with ‘herd immunity’ is quite uncertain due to the emergence of COVID-19 variants [8,9]. Therefore, it is critical to develop effective antiviral drugs that mitigate the lethal cytokine storm in COVID-19 patients.

SARS-CoV-2 belongs to a family of beta-coronaviruses, including SARS-CoV and MERS-CoV, which were responsible for earlier outbreaks of SARS and MERS in 2003 and 2012, respectively [10,11,12]. SARS-CoV-2 encodes two proteases, a 3-chymotrypsin-like cysteine protease (3CLpro) also known as main protease (Mpro) and a papain-like protease (PLpro) for proteolytic processing of viral replication and maturation. [13,14] Both of these proteases are essential for replication of SARS-CoV-2 and other coronaviruses. The SARS-CoV-2 genome shares 80% nucleotide identity with SARS-CoV. Structurally, 3CLpro protease of SARS-CoV-2 shares more than 90% amino acid sequence identity with highly conserved substrate-binding sites with in the active site. [15,16] Thus, previous medicinal chemistry efforts leading to the development of 3CL protease inhibitors of SARS-CoV and MERS-CoV provided important groundwork for drug design efforts against COVID-19 [17,18,19].

**Figure 1:**
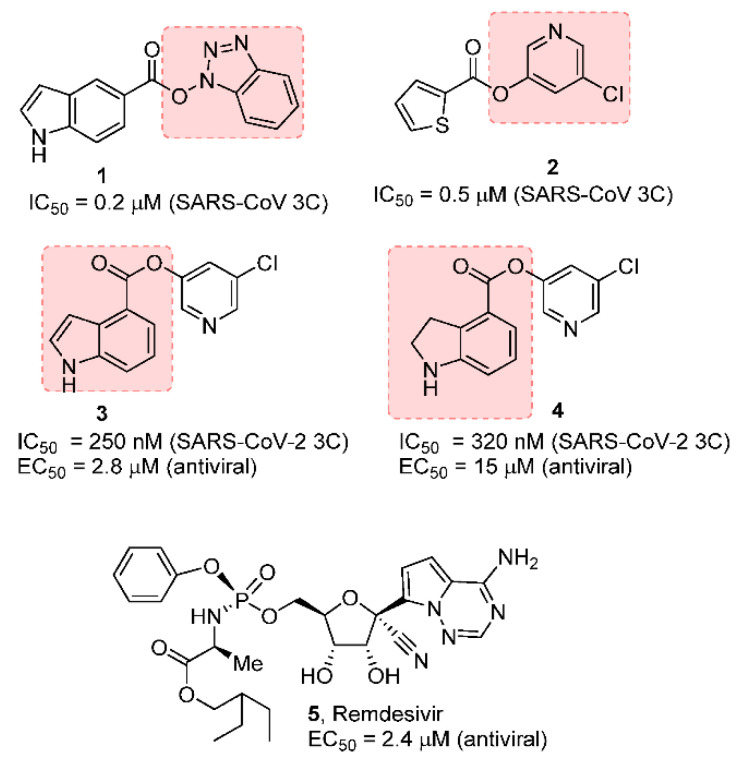
Covalent SARS-CoV and SARS-CoV-2 3CL inhibitors **1**–**4** and remdesivir.

Our laboratories previously designed, synthesized, and performed X-ray structural studies of a variety of peptidomimetic and nonpeptide, covalent, noncovalent MERS-CoV 3CLpro inhibitors that show potent enzyme inhibitory activity and exerted significant antiviral activity against SARS-CoV. [17,20] In particular, we and others have designed a new class of potent and small-molecule covalent SARS-CoV inhibitors that involve acylation of the active site Cys145, forming a covalent bond with the small-molecule inhibitors [21,22,23,24]. As shown in Figure 1, the indole carboxylic acid-derived benzotriazole ester **1** and thiophene carboxylic acid-derived 5-chloropyridinyl ester **2** are potent against SARS-CoV 3CL protease. However, these compounds did not show antiviral activity.

We subsequently designed a series of 5-chloropyridin-3-yl esters and demonstrated both enzyme inhibitory and antiviral activity. Compound **3** exhibited a SARS-CoV 3CL protease inhibitory IC_50_ value of 250 nM and an antiviral EC_50_ value of 2.8 µM in VeroE6 cells [25]. Based upon our previous results, we recently developed indole chloropyridin-3-yl ester-derived SARS-CoV-2 3CL protease inhibitors and demonstrated that prototype compounds, such as inhibitor **3**, are potent inhibitors of SARS-CoV-2 3CL protease. It exerts comparable antiviral activity to remdesivir (**5**), an RNA-dependent RNA-polymerase inhibitor [26,27]. Furthermore, we have shown that compound **3** blocked the infectivity and cytopathic effect of SARS-CoV-2^wk-521^ in VeroE6 cells in our immunocytochemistry assay.

**Figure 2:**
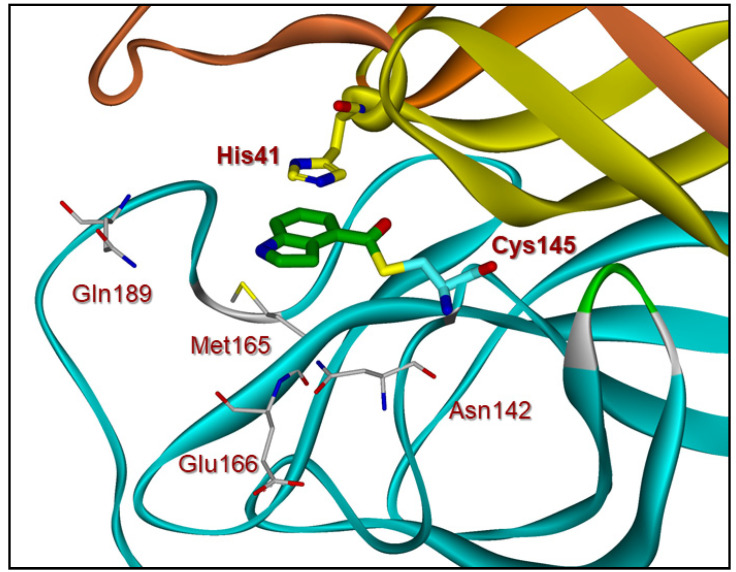
X-ray crystal structure of inhibitor **4**-bound SARS-CoV-2 3CL protease (PDB code:7RBZ). The thioester bond between Cys145 and compound **4** (green) is shown.

Compound **4** has also shown potent enzyme inhibitory and antiviral activity in our immunocytochemistry assays [28]. Our X-ray structural analysis of inhibitor **4** and SARS-CoV-2 3CL protease complex demonstrates that the mode of inhibition involved the formation of a covalent bond with the inhibitor carbonyl group and catalytic Cys145 in the active site as shown in Figure 2. Furthermore, our recent structure–activity relationship (SAR) suggested that the position of the carboxylic acid on the indole scaffold is critical for the enzyme activity. Based upon our X-ray structural studies and mode of inhibition, we have further investigated other aromatic and heteroaromatic scaffolds and their ability to block SARS-CoV-2 3CL protease activity as well as antiviral activity in VeroE6 cells [29]. In particular, we plan to synthesize 5-chloropyridin-3-yl esters of widely used nonsteroidal anti-inflammatory agents (NSAIDs) [30,31] and evaluate their potential as irreversible inhibitors of SARS-CoV-2 3CLpro enzyme. Presumably, such acylated thioesters of these NSAIDs would hydrolyze slowly over time and release parent NSAIDs in the cell [32]. Interestingly, this would lead to inhibition of cyclooxygenase in the cell, leading to analgesic and anti-inflammatory effects. In the present studies, we report a series of 5-chloropyridinyl ester derivatives of salicylic acid, ibuprofen, naproxane, indomethacin, and related interesting derivatives. A number of compounds exhibited potent enzyme inhibitory and antiviral activity.

## 2. Results and Discussion

### 2.1. Chemistry

**Scheme 1:**
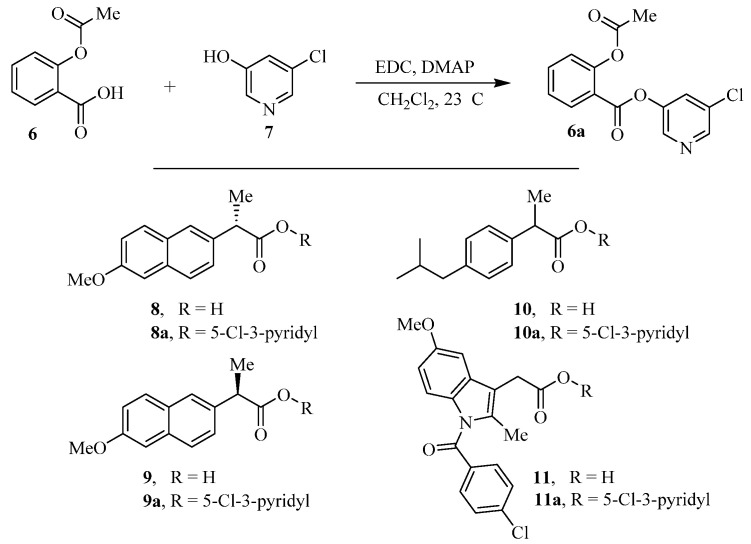
Synthesis of 5-chloropyridin-3-yl esters of common NSAIDs.

**Figure 3:**
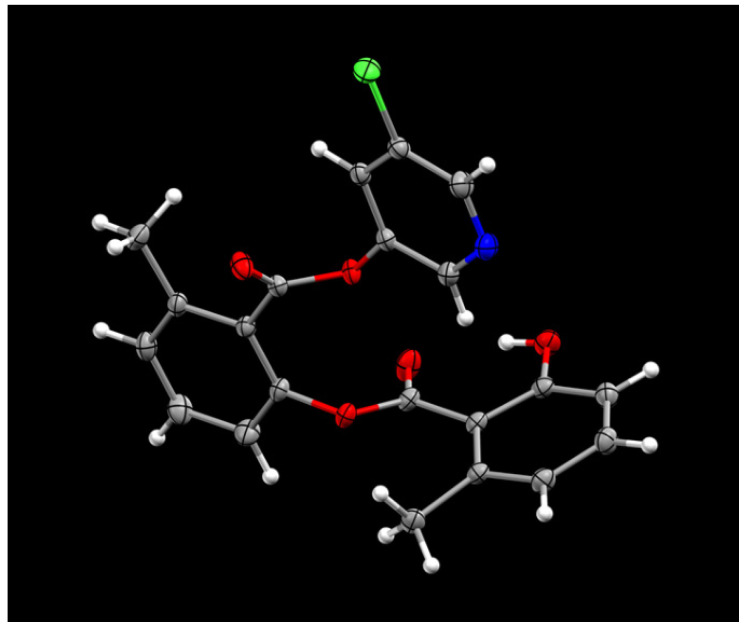
ORTEP drawing of compound **16b** at 50% probability level.

**Scheme 2:**
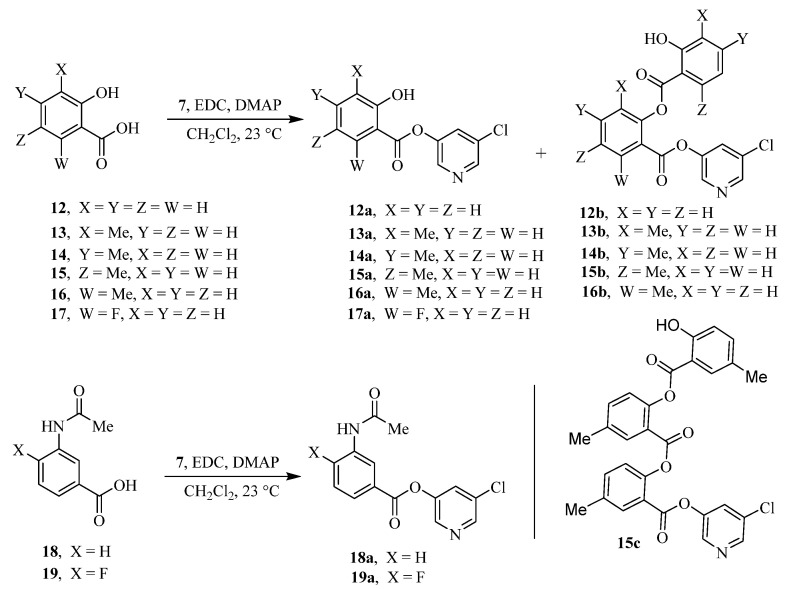
Synthesis of chloropyridinyl esters of salicylic acid derivatives.

The synthesis of various 5-chloropyridinyl esters of common nonsteroidal anti-inflammatory agents [30,31] is shown in Scheme 1. Commercially available aspirin **6** was esterified with 1.2 equivalents of 5-chloro-3-pyridinol using 1.5 equivalents of EDC in the presence of 1 equivalent of DMAP in CH_2_Cl_2_ at 23 °C for 12 h. This condition has provided **6a** in 12% yield after silica gel chromatography, we have then exposed (*S*)- and (*R*)-naproxen, racemic ibuprofen and indomethacin under these esterification conditions for the synthesis of other 5-chloropyridinyl esters **8a**–**11a** (46–53% yield). The structures of these ester derivatives are shown in Table 1. We have also prepared chloropyridinyl esters derived from salicylic acid and its methyl-substituted derivatives as shown in Scheme 2. Initially, we attempted the synthesis of salicylic acid derivatives by using conditions mentioned above. However, the above conditions provided variable results and provided mixture of monomeric and dimeric ester along with small amounts of higher oligomers. The monomeric products were variable. We then carried out the esterification reaction of salicylic acid and its methyl-substituted derivatives by first exposing 0.25 equivalent of acid, 1.2 equivalent of 3-hydroxy-5-chloro-pyridine and 1.5 equivalent of EDC in the presence of 1.0 equivalent DMAP at 23 °C. The mixture was stirred for 2 h and then 0.25 equivalent of acid was added at 23 °C every 2 h interval. The resulting mixture was stirred at 23 °C for 12 h. This condition provided a mixture of monomeric esters (**12a**–**17a**) and dimeric esters (**12b**–**16b**) respectively, in good yields. In the case of 2-hydroxy-5-methyl benzoic acid **15**, in addition to monomer **15a** and dimer derivative **15b**, we have also obtained triester derivative **15c**. 2-Hydroxy-6-methyl benzoic acid **16** provided monoester **16a** and diester **16b**. The diester **16b** was crystalized in CH_2_Cl_2_ solution and the identity of the structure was unambiguously determined by X-ray crystallography as depicted in Figure 3 [33,34]. Please see Supporting Information for further details. For our structure–activity relationship studies, we have also prepared 3-acetamido-benzoic acid ester derivatives **18a** and **19a** in good yield.

### 2.2. Biological Evaluation

We have carried out SARS-CoV-2 3CLpro inhibition assays using the authentic SARS-CoV-2 3CLpro enzyme as described recently [35]. The enzyme inhibitory activity (IC_50_ values) of synthetic active esters was assessed using a continuous fluorescence assay and the FRET-based substrate UIVT3 (HiLyteFluor_488_^TM^_-_EATLQSGLRKAK-QXL_520_-NH_2_ (HPLC > 90%); Anaspec, Fremont, CA, USA) described by us previously [20,36]. The antiviral activity (EC_50_ value) of compounds was evaluated using quantitative VeroE_6_ cell-based assay with RNA-qPCR as described by us recently [28]. The structures and activity of synthetic ester derivatives are shown in Table 1 and Table 2. We first assessed common NSAIDs-derived chloropyridinyl esters shown in Table 1. As can be seen, acetoxysalicylic acid-derived ester **6a** exhibited SARS-CoV-2 3CL protease IC_50_ value of 360 nM (entry 1). This compound exhibited an antiviral EC_50_ value > 100 µM. (*S*)-Naproxen-derived ester **8a** exhibited an enzyme inhibitory activity value of 670 nM (entry 2), while the (*R*)-naproxen derivative **9a** exhibited an enzyme IC_50_ value of 160 nM (entry 3), an over 4-fold improvement. Racemic ibuprofen-derived ester **10a** exhibited an IC_50_ value of 810 nM (entry 4). Indomethacin-derived ester **11a** exhibited a significant reduction in enzyme activity while structurally related indole derivatives exhibited excellent enzyme inhibitory activity (entry 5) [29]. Interestingly, compound **11a** exhibited an antiviral EC_50_ value of 30 µM while aspirin, ibuprofen, and naproxen-derived esters did not show appreciable antiviral activity. We presume that the mode of inhibition involves covalent bond formation with catalytic Cys145 as observed in our previous X-ray structural analysis of inhibitor-bound SARS-CoV-2 3CL protease as well as mass spectral analysis. [20,29] The irreversible enzyme acylation of the NSAIDs-based inhibitor was examined by using MALDI-TOF. Authentic SARS-CoV-2 3CLpro was incubated with inhibitor **8a** and then analyzed with untreated enzyme. As expected, we were able to see a signal for enzyme-bound compound **8a** on the LC–MS spectrum that corresponds to acylation of 3CL protease with a mass shift of +212 Daltons.

Based upon the encouraging enzyme inhibitory activity of NSAIDS derivatives, we then prepared a range of salicylic acid derivatives and evaluated their activity. As shown in Table 2, salicylic acid-derived pyridinyl ester **12a** exhibited an IC_50_ value of 3.47 µM. The corresponding diester derivative **12b** exhibited a 5-fold reduction in enzyme activity. However, compound **12b** exhibited an antiviral EC_50_ value of 64 µM (entry 2). In an effort to modulate activity, we incorporated the methyl group on the aromatic ring. Methyl substitution at C3 resulted in monoester **13a**, which exhibited a significant improvement in enzyme activity (IC_50_ 650 nM) over its unsubstituted derivative **12a**. Diester derivative **13b** exhibited a reduction in the enzyme IC_50_ value but some improvement in antiviral activity (entries 3 and 4). Incorporation of methyl group at C4 provided slight improvement in enzyme activity for both mono- and di-ester derivatives **14a** and **14b** (entries 5 and 6). Substitution of methyl group at C5 led to the syntheses of mono-ester **15a**, diester **15b**, and tri-ester **15c**. Both mono-ester **15a** and diester **15b** exhibited improvement in enzyme activity over other substituted derivatives (entries 7–9). Interestingly, substitution of methyl group at C6 resulted in significant loss of enzyme activity (entries 10 and 11). Incorporation of fluorine at C6 also resulted in a further reduction in enzyme inhibitory activity (entry 12). We have also investigated amide derivatives **18a** and **19a**. Both compounds exhibited enzyme inhibitory activity in low micromolar range (entries 13 and 14). All compounds in Table 1 and Table 2 exhibited a cytotoxicity (CC_50_) value >100 µM. While a number of chloropyridinyl esters exhibited low nanomolar 3CLpro inhibitory activity, the majority of these ester derivatives did not show appreciable antiviral activity, except compounds **11a**, **12b**, **13b**, **15a** and **15b**, which exhibited antiviral EC_50_ values of 24–64 µM. Such high ratios of antiviral EC_50_ and enzyme IC_50_ values may be due to the expression of the efflux transporter *P*-glycoprotein in VeroE6 cells. [37,38] We, therefore, examined the antiviral activity of selected compounds (**6a**, **9a**, **11a**, **14a**, and **15a**) in the presence of *P*-glycoprotein inhibitor, CP-100356 [39]. Interestingly, none of these compounds exhibited any significant antiviral activity in the presence of the *P*-glycoprotein inhibitor.

**Figure 4:**
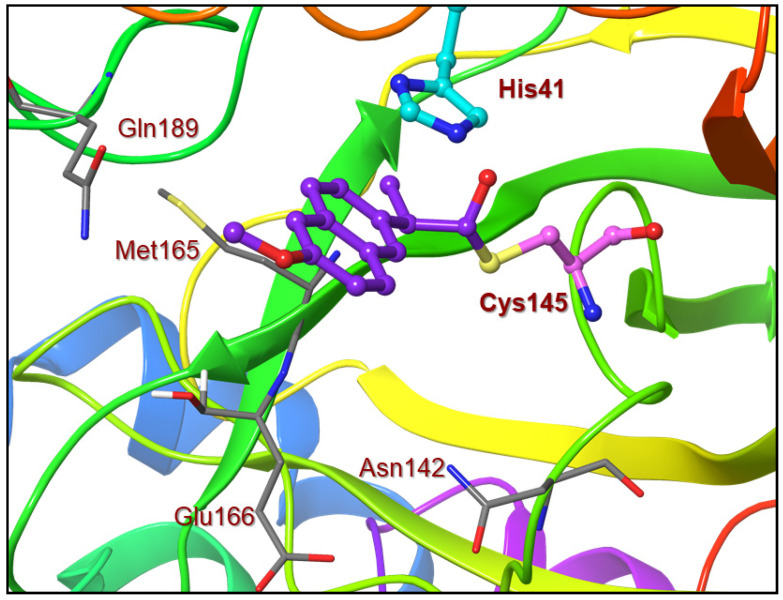
An optimized model of inhibitor **9a** (violet) in the active site of SARS-CoV-2 3CL protease. The model was created based upon the X-ray structure of inhibitor 4 bound to SARS-CoV-2 3CL protease (PDB code:7RBZ).

Based upon the X-ray structure of an irreversible inhibitor (GRL-017-20) bound to SARS-CoV-2 3CL protease (PDB code: 7RBZ), we modeled the complex of inhibitor **9a** with SARS-CoV-2 3CL protease [29]. The model of inhibitor **9a** bound to the catalytic Cys 145 residue in the active site of the 3CL protease is shown in Figure 4. The sulfur atom of Cys145 forms a covalent bond to the carbonyl and the chloropyridinyl group acts as a leaving group. The ligand sits in the binding pocket formed from Asn142, Met165, Glu166 and Gln 189. The model shows a similar π–π stacking of the aromatic ring with the imidazole ring of the His 41 residue [29]. Unfortunately, we did not observe the anticipated hydrogen bond between the methoxy group oxygen atom of the ligand and the side chain of the Gln 189 residue. The current study can provide a foundation to design new irreversible inhibitors to target SARS-CoV-2 3CL protease. Our laboratory is actively working on the design and synthesis of potent COVID-19 inhibitors.

## 3. Materials and Methods

### 3.1. Chemistry

All compounds were purified by column chromatography. Column chromatography was performed using silica gel 230–400 mesh, with a 60 Å pore diameter. Proton Nuclear Magnetic Resonance NMR (^1^H NMR) spectra and carbon nuclear magnetic resonance (^13^C NMR) spectra were recorded on Bruker AV-III-400HD and Bruker AVIII-800 spectrometers. Optical rotations were measured on a Rudolph’s AUTOPOL-III automatic digital polarimeter with a sodium lamp and are reported as follows: [α]λ T °C (*c* = g/100 mL, solvent). High-resolution mass spectrometry (HRMS) spectra were recorded under positive electron spray ionization (ESI+) using a LTQ Orbitrap Mass Spectrometer at the Purdue University Department of Chemistry Mass Spectrometry Center and an Agilent 6550 Q-TOF LC/MS instrument at the Purdue University Analytical Mass Spectrometry Facility.

X-ray diffraction data for the single crystal of compound **16b** were collected on a Bruker Quest diffractometer. Examination and data collection were performed with Mo Kα radiation (λ = 0.71073 Å) at 150 K. Data were collected, reflections were indexed and processed, and the files scaled and corrected for absorption using APEX3 [40]. The space groups were assigned, and the structures were solved by direct methods using XPREP within the SHELXTL suite of programs [41,42] and refined by full-matrix least squares against F2 with all reflections using Shelxl2018 using the graphical interface Shelxle [43]. Complete crystallographic data, in CIF format, have been deposited with the Cambridge Crystallographic Data Centre. The details of the structure are provided in the Supporting Information Section.

Synthesis of 6-chloropyridin-2-yl 2-acetoxybenzoate (6a). To a stirred solution of 2-acetoxybenzoic acid (150 mg, 0.83 mmol) in DCM (3 mL), 5-chloropyridin-3-ol (129.4 mg, 0.10 mmol), EDC (240 mg, 1.25 mmol) and DMAP (102 mg, 0.83 mmol) were added. The resulting reaction mixture was stirred at 23 °C for 10 h. After this period, the reaction mixture was washed with saturated aqueous NaHCO_3_. The organic layer was dried over anhydrous Na_2_SO_4_ and concentrated under reduced pressure to give a crude residue. The residue was purified via silica gel column chromatography (30% ethyl acetate in hexanes) to afford the title ester **6a** (30 mg, 12%) as an amorphous solid. ^1^H NMR (400 MHz, CDCl_3_) δ 8.51 (d, *J* = 2.1 Hz, 1H), 8.42 (d, *J* = 2.4 Hz, 1H), 8.20 (dd, *J* = 7.9, 1.7 Hz, 1H), 7.68 (ddd, *J* = 8.1, 7.5, 1.7 Hz, 1H), 7.64 (t, *J* = 2.2 Hz, 1H), 7.41 (td, *J* = 7.7, 1.2 Hz, 1H), 7.20 (dd, *J* = 8.1, 1.2 Hz, 1H), 2.32 (s, 3H); ^13^C NMR (100 MHz, CDCl_3_) δ 169.5, 161.9, 151.4, 146.1, 145.7, 141.3, 135.2, 132.0, 129.6, 129.4, 126.2, 124.1, 121.3, 20.9; LRMS-ESI (m/z): 292.0 [M + H]^+^. HRMS (ESI/LTQ) *m*/*z*. [M + H]^+^ calcd for C_14_H_11_ClNO_4_ 292.03711; found 292.03630.
5-chloropyridin-3-yl (S)-2-(6-methoxynaphthalen-2-yl)propanoate (8a). Commercially available (S)-2-(6-methoxynaphthalen-2-yl)propanoic acid (50 mg, 0.22 mmol) was esterified with 5-chloropyridin-3-ol (34 mg, 0.26 mmol) by following the procedure for ester **6a** to provide the title ester **8a** (36 mg, 49%) as an amorphous solid. ^1^H NMR (400 MHz, CDCl_3_) δ 8.42–8.40 (m, 1H), 8.25–8.23 (m, 1H), 7.78–7.72 (m, 3H), 7.48–7.41 (m, 2H), 7.20–7.13 (m, 2H), 4.11 (q, *J* = 7.1 Hz, 1H), 3.93 (s, 3H), 1.70 (d, *J* = 7.1 Hz, 3H); ^13^C NMR (100 MHz, CDCl_3_) δ 172.25, 157.8, 147.2, 145.6, 140.9, 134.1, 133.8, 131.6, 129.2, 128.8, 127.5, 126.1, 125.7, 119.2, 105.5, 55.2, 45.4, 18.2; LRMS-ESI (m/z): 342.0 [M + H]^+^. HRMS (ESI/LTQ) *m*/*z*. [M + H]^+^ calcd for C_19_H_17_ClNO_3_ 342.08915; found 342.08907.
5-chloropyridin-3-yl (R)-2-(6-methoxynaphthalen-2-yl)propanoate (9a). Commercially available (R)-2-(6-methoxynaphthalen-2-yl)propanoic acid (60 mg, 0.26 mmol) was esterified with 5-chloropyridin-3-ol (40 mg, 0.31 mmol) by following the procedure for ester 6a to provide the title ester **9a** (45 mg, 50%) as an amorphous solid. ^1^H NMR (400 MHz, CDCl_3_) δ 8.42 (d, *J* = 2.1 Hz, 1H), 8.24 (d, *J* = 2.3 Hz, 1H), 7.79–7.72 (m, 3H), 7.49–7.42 (m, 2H), 7.18 (dd, *J* = 8.9, 2.5 Hz, 1H), 7.14 (d, *J* = 2.5 Hz, 1H), 4.12 (q, *J* = 7.1 Hz, 1H), 3.92 (s, 3H), 1.70 (d, *J* = 7.1 Hz, 3H); ^13^C NMR (100 MHz, CDCl_3_) δ 172.2, 157.8, 147.2, 145.8, 141.1, 134.2, 133.9, 131.5, 129.2, 129.1, 128.9, 127.5, 126.1, 125.7, 119.3, 105.5, 55.2, 45.4, 18.3; LRMS-ESI (*m*/*z*): 342.1 [M + H]^+^.
5-chloropyridin-3-yl 2-(4-isobutylphenyl)propanoate (10a). Commercially available 2-(4-isobutylphenyl)propanoic acid (50 mg, 0.24 mmol) was esterified with 5-chloropyridin-3-ol (47 mg, 0.36 mmol) by following the procedure for ester **6a** to provide the title ester **10a** (35 mg, 46%) as an amorphous solid. ^1^H NMR (400 MHz, CDCl_3_) δ 8.43–8.41 (m, 1H), 8.25–8.22 (m, 1H), 7.44 (t, *J* = 2.2 Hz, 1H), 7.29–7.25 (m, 2H), 7.15 (d, *J* = 8.1 Hz, 2H), 3.95 (q, *J* = 7.1 Hz, 1H), 2.47 (d, *J* = 7.2 Hz, 2H), 1.93–1.81 (m, 1H), 1.61 (d, *J* = 7.1 Hz, 3H), 0.91 (d, *J* = 6.6 Hz, 6H); ^13^C NMR (100 MHz, CDCl_3_) δ 172.3, 147.3, 145.7, 141.1, 141.1, 136.3, 131.6, 129.6, 129.2, 127.1, 45.1, 44.9, 30.1, 22.3, 18.3; LRMS-ESI (*m*/*z*): 318.1 [M + H]^+^. HRMS (ESI/LTQ) *m*/*z*. [M + H]^+^ calcd for C_18_H_21_ClNO_2_ 318.1255; found 318.1251.
5-chloropyridin-3-yl 2-(1-(4-chlorobenzoyl)-5-methoxy-2-methyl-1H-indol-3-yl)acetate (11a). Commercially available 2-(1-(4-chlorobenzoyl)-5-methoxy-2-methyl-1H-indol-3-yl)acetic acid (50 mg, 0.14 mmol) was esterified with 5-chloropyridin-3-ol (22 mg, 0.17mmol) by following the procedure for ester **6a** to provide the title ester **11a** (36 mg, 53%) as an amorphous solid. ^1^H NMR (400 MHz, CDCl_3_) δ 8.39 (d, *J* = 53.8 Hz, 2H), 7.68 (d, *J* = 8.2 Hz, 2H), 7.56–7.43 (m, 3H), 7.01 (d, *J* = 2.6 Hz, 1H), 6.87 (d, *J* = 9.0 Hz, 1H), 6.70 (dd, *J* = 9.0, 2.5 Hz, 1H), 3.94 (s, 2H), 3.84 (s, 3H), 2.46 (s, 3H); ^13^C NMR (100 MHz, CDCl_3_) δ 168.3, 168.2, 156.1, 145.9, 141.0, 139.4, 136.4, 133.6, 131.1, 130.7, 130.1, 129.2, 129.1, 115.0, 111.7, 111.0, 101.1, 55.7, 30.3, 13.3; LRMS-ESI (*m*/*z*): 470.1 [M + H]^+^. HRMS (ESI/LTQ) *m*/*z*. [M + H]^+^ calcd for C_24_H_19_Cl_2_N_2_O_4_ 469.07164; found 469.07131.
5-chloropyridin-3-yl 2-hydroxybenzoate (12a) and 5-chloropyridin-3-yl 2-((2-hydroxybenzoyl)oxy)benzoate (12b). Commercially available 2-hydroxybenzoic acid (50 mg, 0.36 mmol) was esterified with 5-chloropyridin-3-ol (56.3 mg, 0.43mmol) by following the procedure for ester **6a** to provide the title monoester **12a** (10 mg, 11%), and diester **12b** (13 mg, 10%) as amorphous solid.
**12a:** ^1^H NMR (400 MHz, CDCl_3_) δ 10.17 (d, *J* = 0.5 Hz, 1H), 8.54 (dd, *J* = 2.0, 0.5 Hz, 1H), 8.48 (dd, *J* = 2.3, 0.5 Hz, 1H), 8.04 (ddd, *J* = 8.0, 1.8, 0.5 Hz, 1H), 7.68 (t, *J* = 2.2 Hz, 1H), 7.58 (dddd, *J* = 8.4, 7.2, 1.7, 0.5 Hz, 1H), 7.07 (ddd, *J* = 8.5, 1.1, 0.5 Hz, 1H), 7.03–6.98 (m, 1H); ^13^C NMR (100 MHz, CDCl_3_) δ 167.9, 162.4, 146.4, 141.3, 137.2 (×2), 131.9, 130.2, 129.5, 119.7, 118.1, 110.8; LRMS-ESI (*m*/*z*): 250.0 [M + H]^+^. HRMS (ESI/LTQ) *m*/*z*. [M + H]^+^ calcd for C_12_H_9_ClNO_3_ 250.02655; found 250.02579.
**12b:**^1^H NMR (400 MHz, CDCl_3_) δ 10.24 (d, *J* = 0.4 Hz, 1H), 8.44 (dd, *J* = 2.1, 0.5 Hz, 1H), 8.32–8.26 (m, 2H), 8.09 (ddd, *J* = 8.0, 1.8, 0.4 Hz, 1H), 7.76 (ddd, *J* = 8.2, 7.5, 1.7 Hz, 1H), 7.55–7.48 (m, 3H), 7.35 (dd, *J* = 8.2, 1.1 Hz, 1H), 7.02 (ddd, *J* = 8.5, 1.1, 0.4 Hz, 1H), 6.96 (ddd, *J* = 8.3, 7.3, 1.1 Hz, 1H); ^13^C NMR (200 MHz, CDCl_3_) δ 168.7, 162.1, 161.7, 150.5, 146.9, 146.1, 141.1, 136.7, 135.3, 132.4, 131.8, 130.4, 129.4, 126.8, 124.3, 121.7, 119.7, 117.9, 111.5; LRMS-ESI (*m*/*z*): 370.0 [M + H]^+^. HRMS (ESI/LTQ) m/z. [M + H]^+^ calcd for C19H13ClNO5 370.04768; found 370.04688.
5-chloropyridin-3-yl 2-hydroxy-3-methylbenzoate (13a) and 5-chloropyridin-3-yl 2-((2-hydroxy-3-methylbenzoyl)oxy)-3-methylbenzoate (13b). Commercially available 2-hydroxy-3-methylbenzoic acid (30 mg, 0.20 mmol) was esterified with 5-chloropyridin-3-ol (31.0 mg, 0.24 mmol) by following the procedure for ester **6a** to provide the title monoester **13a** (10 mg, 19%), and diester **13b** (17 mg, 22%) as amorphous solid.
**13a:** ^1^H NMR (400 MHz, CDCl_3_) δ 10.41 (s, 1H), 8.51 (d, *J* = 27.8 Hz, 2H), 7.89 (d, *J* = 8.0 Hz, 1H), 7.71–7.63 (m, 1H), 7.44 (d, *J* = 7.2 Hz, 1H), 6.89 (t, *J* = 7.7 Hz, 1H), 1.56 (s, 3H).; ^13^C NMR (200 MHz, CDCl_3_) δ 168.3, 160.8, 146.2, 141.2, 137.9 (2), 129.7, 127.7, 127.2, 119.1 (2), 110.0, 15.6; LRMS-ESI (m/z): 264.0 [M + H]^+^. HRMS (ESI/LTQ) *m*/*z*. [M + H]^+^ calcd for C_13_H_11_ClNO_3_ 264.04220; found 264.04152.
**13b:** ^1^H NMR (400 MHz, CDCl_3_) δ 10.55 (s, 1H), 8.42 (s, 1H), 8.30 (s, 1H), 8.13–8.05 (m, 1H), 8.00–7.92 (m, 1H), 7.61 (d, *J* = 7.5 Hz, 1H), 7.54–7.47 (m, 1H), 7.44–7.33 (m, 2H), 6.86 (t, *J* = 7.7 Hz, 1H), 2.32 (s, 3H), 2.28 (s, 3H).; ^13^C NMR (200 MHz, CDCl_3_) δ 168.8, 162.0, 160.5, 149.1, 145.9, 141.1, 137.5, 136.9, 132.9, 130.1, 129.5, 127.9 (2), 127.0, 126.4 (2), 121.7, 119.1, 110.6, 16.2, 15.6; LRMS-ESI (m/z): 398.0 [M + H]^+^. HRMS (ESI/LTQ) *m*/*z*. [M + H]^+^ calcd for C_21_H_17_ClNO_5_ 398.07898; found 398.07824.
5-chloropyridin-3-yl 2-hydroxy-4-methylbenzoate (14a) and 5-chloropyridin-3-yl 2-((2-hydroxy-4-methylbenzoyl)oxy)-4-methylbenzoate (14b). Commercially available 2-hydroxy-4-methylbenzoic acid (200 mg, 1.31 mmol) was esterified with 5-chloropyridin-3-ol (204.3 mg, 1.58 mmol) by following the procedure for ester **6a** to provide the title monoester **14a** (52 mg, 15%), and diester **14b** (15 mg, 3%) as amorphous solid.
**14a:** ^1^H NMR (400 MHz, CDCl_3_) δ 10.11 (s, 1H), 8.52 (d, *J* = 2.1 Hz, 1H), 8.46 (d, *J* = 2.4 Hz, 1H), 7.89 (d, *J* = 8.2 Hz, 1H), 7.66 (t, *J* = 2.2 Hz, 1H), 6.86 (dd, *J* = 1.8, 0.9 Hz, 1H), 6.83–6.76 (m, 1H), 2.39 (s, 3H); ^13^C NMR (100 MHz, CDCl_3_) δ 167.7, 162.3, 148.9, 146.7, 146.2, 141.3, 131.8, 130.0, 129.5, 121.0, 118.1, 108.1, 22.0; LRMS-ESI (*m*/*z*): 264.0 [M + H]^+^. HRMS (ESI/LTQ) *m*/*z*. [M + H]^+^ calcd for C_13_H_11_ClNO_3_ 264.04220; found 264.04148.
**14b:** ^1^H NMR (400 MHz, CDCl_3_) δ 10.21 (s, 1H), 8.42 (d, *J* = 2.1 Hz, 1H), 8.30 (d, *J* = 2.3 Hz, 1H), 8.14 (d, *J* = 8.0 Hz, 1H), 7.95 (d, *J* = 8.1 Hz, 1H), 7.51 (t, *J* = 2.2 Hz, 1H), 7.28 (ddd, *J* = 8.1, 1.7, 0.8 Hz, 1H), 7.14 (d, *J* = 0.8 Hz, 1H), 6.82 (dd, *J* = 1.8, 0.9 Hz, 1H), 6.76 (ddd, *J* = 8.1, 1.6, 0.6 Hz, 1H), 2.50 (s, 3H), 2.36 (s, 3H); ^13^C NMR (200 MHz, CDCl_3_) δ 168.8, 162.0, 161.8, 150.5, 148.3, 147.0, 147.0, 145.9, 141.1, 132.3, 131.7, 130.2, 129.5, 127.6, 124.8, 121.0, 118.8, 117.9, 109.0, 21.9, 21.6; LRMS-ESI (*m*/*z*): 398.0 [M + H]^+^. HRMS (ESI/LTQ) *m*/*z*. [M + H]^+^ calcd for C_21_H_17_ClNO_5_ 398.07898; found 398.07865.
5-chloropyridin-3-yl 2-hydroxy-5-methylbenzoate (15a) and 5-chloropyridin-3-yl 2-((2-hydroxy-5-methylbenzoyl)oxy)-5-methylbenzoate (15b) and 6-chloropyridin-2-yl 2-((2-((2-hydroxy-5-methylbenzoyl)oxy)-5-methylbenzoyl)oxy)-5-methylbenzoate (15c). Commercially available 2-hydroxy-5-methylbenzoic acid (200 mg, 1.31 mmol) was esterified with 5-chloropyridin-3-ol (204.3 mg, 1.58 mmol) by following the procedure for ester **6a** to provide the title monoester **15a** (72 mg, 21%), diester **15b** (29 mg, 5%), and triester **15c** (11 mg, 5%) as amorphous solid.
**15a:** ^1^H NMR (400 MHz, CDCl_3_) δ 9.97 (s, 1H), 8.49 (dd, *J* = 24.3, 2.3 Hz, 2H), 7.81 (d, *J* = 2.4 Hz, 1H), 7.66 (t, *J* = 2.2 Hz, 1H), 7.37 (dd, *J* = 8.6, 2.3 Hz, 1H), 6.95 (d, *J* = 8.5 Hz, 1H), 2.33 (s, 3H); ^13^C NMR (100 MHz, CDCl_3_) δ 167.8, 160.3, 146.7, 146.3, 141.3, 138.2, 131.8, 129.7, 129.5, 129.0, 117.8, 110.3, 20.3; LRMS-ESI (*m*/*z*): 264.0 [M + H]^+^. HRMS (ESI/LTQ) *m*/*z*. [M + H]^+^ calcd for C_13_H_11_ClNO_3_ 264.04220; found 264.04128.
**15b:**^1^H NMR (400 MHz, CDCl_3_) δ 10.09 (s, 1H), 8.43 (s, 1H), 8.31 (s, 1H), 8.05 (d, *J* = 2.4 Hz, 1H), 7.87 (d, *J* = 2.4 Hz, 1H), 7.53 (dt, *J* = 4.4, 2.0 Hz, 2H), 7.35–7.19 (m, 2H), 6.91 (d, *J* = 8.5 Hz, 1H), 2.48 (s, 3H), 2.30 (s, 3H); ^13^C NMR (100 MHz, CDCl_3_) δ 168.9, 161.9, 160.0, 148.2, 145.9, 141.0, 137.7, 136.8 (2), 135.92, 132.66, 129.92, 129.53, 128.80, 123.91, 121.18, 117.59 (2), 111.02, 20.77, 20.32.; LRMS-ESI (*m*/*z*): 398.0 [M + H]^+^. HRMS (ESI/LTQ) *m*/*z*. [M + H]^+^ calcd for C_21_H_17_ClNO_5_ 398.07898; found 398.07802.
**15c:**^1^H NMR (400 MHz, CDCl_3_) δ 10.10 (d, *J* = 7.8 Hz, 1H), 8.44 (t, *J* = 3.3 Hz, 1H), 8.32 (d, *J* = 2.6 Hz, 1H), 8.09 (dd, *J* = 2.3, 0.8 Hz, 1H), 7.96 (dd, *J* = 2.2, 0.9 Hz, 1H), 7.84 (dd, *J* = 2.2, 1.0 Hz, 1H), 7.57–7.51 (m, 2H), 7.46 (dddd, J = 11.6, 8.3, 2.3, 0.8 Hz, 2H), 7.16 (d, *J* = 8.2 Hz, 1H), 7.08 (d, *J* = 8.2 Hz, 1H), 6.87 (d, *J* = 8.5 Hz, 1H), 2.43 (s, 3H), 2.41 (s, 3H), 2.25 (s, 3H); ^13^C NMR (100 MHz, CDCl_3_) δ 168.8, 162.9, 159.8, 148.6, 148.2, 145.8, 137.3 (2), 136.6, 136.4, 135.9, 135.4 (2), 132.6, 132.5 (2), 130.1 (2), 129.7, 128.6, 123.8, 123.7, 121.1, 117.3 (2), 111.3, 20.8, 20.7, 20.3; LRMS-ESI (*m*/*z*): 532.0 [M + H]^+^. HRMS (ESI/LTQ) *m*/*z*. [M + H]^+^ calcd for C_29_H_23_ClNO_7_ 532.11576; found 532.11478.
5-chloropyridin-3-yl 2-hydroxy-6-methylbenzoate (16a) and 5-chloropyridin-3-yl 2-((2-hydroxy-6-methylbenzoyl)oxy)-6-methylbenzoate (16b). Commercially available 2-hydroxy-6-methylbenzoic acid (70 mg, 0.46 mmol) was esterified with 5-chloropyridin-3-ol (72 mg, 0.55 mmol) by following the procedure for ester **6a** to provide the title monoester **16a** (14 mg, 12%), and diester **16b** (15 mg, 8%) as amorphous solid.
**16a:** ^1^H NMR (400 MHz, CDCl_3_) δ 10.67 (s, 1H), 8.55 (d, J = 2.1 Hz, 1H), 8.46 (d, *J* = 2.4 Hz, 1H), 7.67 (t, *J* = 2.2 Hz, 1H), 7.39 (dd, *J* = 8.4, 7.5 Hz, 1H), 6.92 (ddd, *J* = 8.4, 1.3, 0.6 Hz, 1H), 6.84–6.81 (m, 1H), 2.69 (s, 3H); ^13^C NMR (100 MHz, CDCl_3_) δ 169.4, 163.7, 146.4 (2), 141.4, 141.2, 135.7, 131.9, 129.6, 123.5, 116.1, 110.7, 24.2; LRMS-ESI (*m*/*z*): 264.0 [M + H]^+^. HRMS (ESI/LTQ) *m*/*z*. [M + H]^+^ calcd for C_13_H_11_ClNO_3_ 264.04220; found 264.04109.
**16b:**^1^H NMR (400 MHz, CDCl_3_) δ 10.78 (s, 1H), 8.42 (d, *J* = 2.1 Hz, 1H), 8.21 (d, *J* = 2.4 Hz, 1H), 7.53 (t, *J* = 7.9 Hz, 1H), 7.40–7.27 (m, 3H), 7.16 (ddd, *J* = 8.2, 1.1, 0.6 Hz, 1H), 6.91–6.86 (m, 1H), 6.79 (ddd, *J* = 7.5, 1.3, 0.7 Hz, 1H), 2.66 (s, 3H), 2.59 (s, 3H); ^13^C NMR (100 MHz, CDCl_3_) δ 170.1, 163.6, 163.4, 148.0, 146.1, 141.4, 140.7, 139.5, 135.5, 132.2, 129.1, 129.1, 124.3, 123.4 (2), 120.7 (2), 116.0, 111.0, 24.1, 20.5; LRMS-ESI (*m*/*z*): 398.0 [M + H]^+^. HRMS (ESI/LTQ) *m*/*z*. [M + H]^+^ calcd for C_21_H_17_ClNO_5_ 398.07898; found 398.07808.
5-chloropyridin-3-yl 2-fluoro-6-hydroxybenzoate (17a). Commercially available 2-fluoro-6-hydroxybenzoic acid (50 mg, 0.32 mmol) was esterified with 5-chloropyridin-3-ol (50 mg, 0.38 mmol) by following the procedure for ester 6a to provide the title ester 17a (11 mg, 13%) as an amorphous solid. ^1^H NMR (400 MHz, CDCl_3_) δ 10.64 (s, 1H), 8.52 (dd, *J* = 21.6, 2.2 Hz, 2H), 7.68 (t, *J* = 2.2 Hz, 1H), 7.50 (td, *J* = 8.4, 6.0 Hz, 1H), 6.87 (dt, *J* = 8.5, 1.1 Hz, 1H), 6.72 (ddd, *J* = 11.0, 8.3, 1.1 Hz, 1H); ^13^C NMR (100 MHz, CDCl_3_) δ 163.5, 146.5, 146.4, 141.2, 137.0, 136.8, 131.8, 129.5, 113.8, 107.5, 107.3, 101.4; LRMS-ESI (*m*/*z*): 268.0 [M + H]^+^.
5-chloropyridin-3-yl 3-acetamidobenzoate (18a). Commercially available 3-acetamidobenzoic acid (50 mg, 0.28 mmol) was esterified with 5-chloropyridin-3-ol (43.4 mg, 0.33 mmol) by following the procedure for ester **6a** to provide the title ester **18a** (21 mg, 26%) as an amorphous solid. ^1^H NMR (400 MHz, CDCl_3_) δ 8.50 (d, *J* = 2.1 Hz, 1H), 8.45 (d, *J* = 2.3 Hz, 1H), 8.20 (t, *J* = 2.0 Hz, 1H), 7.96 (dd, *J* = 8.2, 2.1 Hz, 1H), 7.91 (dt, *J* = 7.9, 1.4 Hz, 1H), 7.66 (t, *J* = 2.2 Hz, 1H), 7.58 (s, 1H), 7.48 (t, *J* = 8.0 Hz, 1H), 2.21 (s, 3H); ^13^C NMR (100 MHz, CDCl_3_) δ 169.6, 164.1, 145.6, 141.1, 138.9, 131.9, 129.8 (2), 129.3, 128.6, 125.5 (2), 121.1, 23.9; LRMS-ESI (*m*/*z*): 291.0 [M + H]^+^. HRMS (ESI/LTQ) *m*/*z*. [M + H]^+^ calcd for C_14_H_12_ClN_2_O_3_ 291.05310; found 291.05263.
5-chloropyridin-3-yl 3-acetamido-4-fluorobenzoate (19a). Commercially available 3-acetamido-4-fluorobenzoic acid (50 mg, 0.25 mmol) was esterified with 5-chloropyridin-3-ol (39.4 mg, 0.30 mmol) by following the procedure for ester **6a** to provide the title ester **19a** (70 mg, 90%) as an amorphous solid. ^1^H NMR (400 MHz, MeOD) δ 8.88 (dd, *J* = 7.4, 2.3 Hz, 1H), 8.41 (dd, *J* = 10.6, 2.2 Hz, 2H), 7.91 (ddd, *J* = 8.6, 4.8, 2.3 Hz, 1H), 7.72 (s, 1H), 7.22 (dd, *J* = 10.3, 8.6 Hz, 1H), 2.19 (s, 3H); ^13^C NMR (100 MHz, MeOD) δ 170.4, 163.3, 147.6, 145.5, 141.0, 132.0, 130.1, 127.6, 127.5, 125.7, 124.4, 115.8, 115.6, 23.2; LRMS-ESI (*m*/*z*): 309.0 [M + H]^+^. HRMS (ESI/LTQ) *m*/*z*. [M + H]^+^ calcd for C_14_H_11_ClFN_2_O_3_ 309.04367; found 309.04413.

### 3.2. IC_50_ Value Determination

IC_50_ values were determined for compounds that covalently inhibit SARS-CoV-2 3CLpro using our recently described assay [28] and data fitting methods that were derived from our previous work on SARS-CoV 3CLpro and inhibition by chloropyridyl esters [22]. The only differences were that pre-incubation of the enzyme with the compounds was 10 min instead of 20 min. In addition, the Morrison Equation was only used to determine the IC_50_ values when they were below 1 µM.

### 3.3. Mass Analysis of Enzyme-Inhibitor Complex

Purified SARS-CoV-2 3CLpro was injected onto a Superdex™ 200 Increase 10/300 GL gel filtration column (GE Healthcare) equilibrated in 20 mM HEPES pH 7.5. Fractions containing pure, active protein were pooled for further analysis. Protein was diluted to a final concentration of 2 μM using 20 mM HEPES pH 7.5 and incubated at room temperature with a final concentration of 20 μM compound **8a**. The protein and ligand were incubated together for ten minutes before analysis.

Analysis of the proteins was performed on a 6550 iFunnel Q-TOF LC/MS (Agilent Technologies, Santa Clara, CA, USA). A sample (6 ul) was injected on to a Zorbax Extend C18 column (Agilent Technologies) kept at 60 degrees C. The mobile phase consisted of B = acetonitrile and A = 0.1% aqueous formic acid. The flow rate was 0.4 mL/min with a gradient as follows: 0–2 min 3% B; 2–7 min 95% B; 7–9 min 95% B; 9–11 min 3% B. For the first 2 min of the analysis, the column flow was diverted off to waste. TOF MS conditions: drying gas temperature 290 degrees C; drying gas flow 14 L/min; sheath gas temperature 400 degrees C; nebulizer 20 psi; capillary voltage 5000 V; nozzle 2000 V.

Positive ion spectra obtained were analyzed using Agilent MassHunter Qualitative Analysis Software B.06 and the deconvolution spectra were generated using Agilent MassHunter BioConfirm software B.06.

### 3.4. Cells, Viruses, and Antiviral Activity

VeroE6 cells and TMPRSS2-overexpressing VeroE6 (VeroE6TMPRSS2) cells were obtained from the Japanese Collection of Research Bioresources (JCRB) Cell Bank (Osaka, Japan). VeroE6 cells were maintained in Dulbecco’s modified Eagle’s medium (d-MEM) supplemented with 10% fetal bovine serum (FCS), 100 µg/mL of penicillin, and 100 µg/mL of streptomycin. VeroE6TMPRSS2 cells were maintained in d-MEM as reported [25] in the presence of 1 mg/mL of G418. SARS-CoV-2 strain JPN/TY/WK-521 (SARS-CoV-2WK-521) was obtained from the National Institute of Infectious Diseases (Tokyo, Japan).

Antiviral assay was carried out as described recently [28]. Cells were seeded in a 96-well plate (2 × 10^4^ cells/well) and incubated. After 24 h, virus was inoculated into cells at multiplicity of infection (MOI) of 0.05. After an additional 72 h, cell culture supernatants were harvested and viral RNA was extracted using a QIAamp viral RNA minikit (Qiagen, Hilden, Germany), and quantitative RT-PCR (RT-qPCR) was then performed using One Step PrimeScript III RT-qPCR mix (TaKaRa Bio, Shiga, Japan) following the instructions of the manufacturers. The primers and probe used for detecting SARS-CoV-2 envelope (6) were 5′-ACT TCT TTT TCT TGC TTT CGT GGT-3′ (forward), 5′-GCA GCA GTA CGC ACA CAA TC-3′ (reverse), and 5′-FAM-CTA GTT ACA CTA GCC ATC CTT ACT GC-black hole quencher 1 (BHQ1)-3′ (probe). To determine the cytotoxicity of each compound, cells were seeded in a 96-well plate (2 × 10^4^ cells/well). One day later, various concentrations of each compound were added, and cells were incubated for an additional 3 days. The 50% cytotoxic concentrations (CC_50_) values were determined using the WST-8 assay and Cell Counting Kit-8 (Dojindo, Kumamoto, Japan).

## 4. Conclusions

In conclusion, we have designed a series of 5-chloropyridinyl esters of common nonsteroidal anti-inflammatory agents. These ester derivatives were synthesized conveniently using EDC in the presence of DMAP to provide stable ester derivatives which were purified by silica gel chromatography. These compounds were evaluated in our in vitro SARS-CoV-2 3CL protease inhibitory assay. A number of compounds exhibited potent low nanomolar enzyme inhibitory activity. Presumably, the mode of inhibition involves the formation of a covalent bond with catalytic Cys145 in the active site. We expanded our studies with salicylic acid-derived derivatives. Methyl group substitution on the aromatic resulted in the synthesis of monomeric, dimeric, and trimeric ester derivatives. Interestingly, methyl substitution led to modulation of enzyme inhibitory activity. A number of these active ester derivatives also exhibited antiviral activity in VeroE_6_ cell-based assays with RNA-qPCR and immunocytochemistry assays. These compounds did not show cytotoxicity up to 100 µM. To obtain molecular insight, we have created an active model of compound **9a**-bound SARS-CoV-2 3CL protease. The model shows formation of a covalent bond with Cys145 and a strong hydrogen bond with Gln189. Active site His41 forms a nice π–π stacking interaction with the napthyl ring of (*R*)-naproxen. Similar π–π stacking interactions were observed in our X-ray crystallographic studies of indol-derived active ester derivatives. Further modification of structures to improve antiviral activity is in progress.

## Figures and Tables

**Table 1:** Structure and activity of NSAIDs-derived chloropyridinyl esters.

No.	Compound Structure	SARS CoV-2 3CLpro IC_50_ (µM)	SARS-CoV-2 EC_50_ (µM) ^a^
1	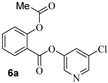	0.36	>100
2	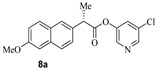	0.67	>100
3	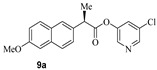	0.16	>100
4	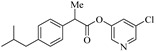	0.81	>100
5	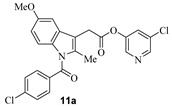	5.32	30.2

^a^ Compounds 3 and 5 exhibited antiviral EC50 values of 2.8 and 2.4 μm, respectively.

**Table 2:** Structure and activity of salicylic acid and its substituted chloropyridinyl esters.

No.	Compound Structure	SARS CoV-2 3CLpro IC_50_ (µM)	SARS-CoV-2 EC_50_ (µM) ^a^
1	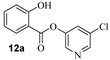	3.47	>100
2	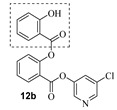	17.1	63.7
3	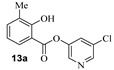	0.65	>100
4	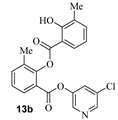	4.9	23.8
5	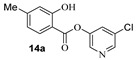	0.54	>100
6	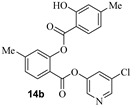	1.4	>100
7	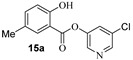	0.39	46.3
8	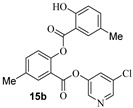	0.74	48.3
9	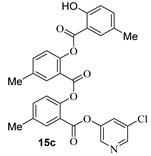	3.73	>100
10	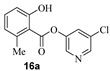	4.26	>100
11	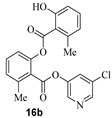	15.4	>100
12	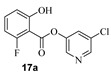	107.8	>100
13	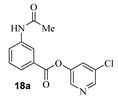	1.11	>100
14	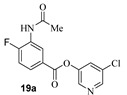	1.51	>100

^a^ Compounds 3 and 5 exhibited antiviral EC50 values of 2.8 and 2.4 μm, respectively.

## Data Availability

Not applicable.

## Supplementary Materials

The following materials are available online. Characterization data, X-ray structural information, and ^1^H-NMR, ^13^C NMR and HRMS data.

## Sample Availability

Samples of the compounds are not available from the authors.
