# Supplementary material for: Chloropyridinyl Esters of Nonsteroidal Anti-Inflammatory Agents and Related Derivatives as Potent SARS-CoV-2 3CL Protease Inhibitors
Source: Molecules. 2021 Sep 24;26(19):5782. doi: 10.3390/molecules26195782 (PMC8510097; doi:10.3390/molecules26195782)
Supplement: Supplementary file 1 [file molecules-26-05782-s001.zip › molecules-1377915-supplementary.pdf]

Supplementary Materials

Chloropyridinyl Esters of Nonsteroidal Anti-Inflammatory Agents and Related Derivatives as Potent SARS-CoV-2 3CL Protease Inhibitors

Arun K. Ghosh [1,*], Dana Shahabi [1], Monika Yadav [1], Satish Kovela [1], Brandon J. Anson [2], Emma K. Lendy [3],
Connie Bonham [4], Devika Sirohi [2,5], Carlos A. Brito-Sierra [2,5], Shin-ichiro Hattori [6,7], Richard Kuhn [2,5], Hiroaki Mitsuya [6,7,8] and Andrew D. Mesecar [2,3]

[1] Department of Chemistry; Department of Medicinal Chemistry, Purdue University, West Lafayette,
IN 47907, USA; dshahabi@purdue.edu (D.S.); yadav21@purdue.edu (M.Y.); satish.kovela@gmail.com (S.K.)
[2] Department of Biological Sciences, Purdue University, West Lafayette, IN 47907, USA; banson@purdue.edu (B.J.A.); dsirohi@purdue.edu (D.S.); cbritosi@purdue.edu (C.A.B.-S.); kuhnr@purdue.edu (R.K.)
[3] Department of Biochemistry, Purdue University, West Lafayette, IN 47907, USA; elendy@purdue.edu (E.K.L.); amesecar@purdue.edu (A.D.M.)
[4] Bindley Bioscience Center, Purdue University, West Lafayette, IN 47907, USA; bonhamc@purdue.edu
[5] Purdue Institute of Inflammation, Immunology and Infectious Disease, Purdue University, West Lafayette, IN 47907, USA
[6] Departments of Hematology and Infectious Diseases, Kumamoto University School of Medicine,
Kumamoto 860-8556, Japan; shattori@ri.ncgm.go.jp (S.-i.H.); hmitsuya@hosp.ncgm.go.jp (H.M.)
[7] Department of Refractory Viral Infections, National Center for Global Health and Medicine Research
Institute, Tokyo 162-8655, Japan
[8] Experimental Retrovirology Section, HIV and AIDS Malignancy Branch, National Cancer Institute,
Bethesda, MD 20892, USA
* Correspondence: akghosh@purdue.edu

Table of Contents

General Methods..S2

X-ray crystallographic data for compound **16b**……..……………………….……..…...S2-S4

Virus and cell biology…………………………………………………………..…….S4-S5

^{1}H- and ^{13}C-NMR Spectra……………………………………………………….....S6-S43

General Methods.

All reactions were carried out under an argon atmosphere in either flame or oven-dried (120 ^oC) glassware. All reagents and chemicals were purchased from commercial suppliers and used without further purification unless otherwise noted. Anhydrous solvents were obtained as follows: Dichloromethane from calcium hydride, methanol and ethanol from activated magnesium under argon. All purification procedures were carried out with reagent grade solvents (purchased form VWR) in air. TLC analysis was conducted using glass-backed Thin-Layer Silica Gel Chromatography Plates (60 Å, 250 µm thickness, F-254 indicator). Column chromatography was performed using 230-400 mesh, 60 Å pore diameter silica gel. ^1H, ^{13}C NMR spectra were recorded at room temperature on a Bruker ARX-400, DRX-500, and Bruker AV800. Chemical shifts (δ values) are reported in parts per million, and are referenced to the deuterated residual solvent peak. NMR data is reported as: δ value (chemical shift, J-value (Hz), integration, where s = singlet, d = doublet, t = triplet, q = quartet, brs = broad singlet). LRMS and HRMS spectra were recorded at the Purdue University Department of Chemistry Mass Spectrometry Center. HPLC analysis and purification was done an on Agilent 1260 series instrument using a YMC Pack ODS-A column of 4.6 mm ID for analysis and either 10 mm ID or 20 mm ID for purification. The purity of all test compounds was determined by HPLC analysis to be ≥90% pure.

X-ray crystallographic data for compound 16b

Single crystals of **16b** were coated with Fomblin oil and transferred to the goniometer head of a Bruker Quest diffractometer with kappa geometry, an I-µ-S microsource X-ray tube, laterally graded multilayer (Goebel) mirror single crystal for monochromatization, a Photon-II CMOS area detector and an Oxford Cryosystems low temperature device. Examination and data collection were performed with Mo Kα radiation (λ =0.71073 Å) at 150 K. Data were collected, reflections were indexed and processed, and the files scaled and corrected for absorption using APEX3[1]. The space groups were assigned, and the structures were solved by direct methods using XPREP within the SHELXTL suite of programs[2,3] and refined by full matrix least squares against F2 with all reflections using Shelxl2018[4] using the graphical interface Shelxle[5]. If not specified otherwise H atoms attached to carbon were positioned geometrically and constrained to ride on their parent atoms. C-H bond distances were constrained to 0.95 Å for aromatic and alkene C-H and CH2 and alkyne C-H moieties, and to 1.00, 0.99 and 0.98 Å for aliphatic C-H, CH2 and CH3 moieties, respectively. Methyl H atoms were allowed to rotate but not to tip to best fit the experimental electron density. $U_{iso}(H)$ values were set to a multiple of $U_{eq}(C)$ with 1.5 for CH3, and 1.2 for C-H and CH2 units, respectively. Additional data collection and refinement details can be found in the Supporting Information. Complete crystallographic data, in CIF format, have been deposited with the Cambridge Crystallographic Data Centre. CCDC 2105273 contain the supplementary crystallographic data for compound **16b**. This data can be obtained free of charge from The Cambridge Crystallographic Data Centre via www.ccdc.cam.ac.uk/data_request/cif. X-ray diffraction data were collected using an instrument funded by the NSF (CHE-1625543).

References:

1. Bruker (2016). Apex3 v2016.9-0, Saint V8.34A, SAINT V8.37A, Bruker AXS Inc.: Madison (WI), USA, 2013/2014.
2. SHELXTL suite of programs, Version 6.14, 2000-2003, Bruker Advanced X-ray Solutions, Bruker AXS Inc., Madison, Wisconsin: USA.
3. Sheldrick, G. *Acta Crystallogr A* **64** (1), 112 (2008).

4. Sheldrick, G. University of Gottingen, Germany, 2018.
5. Sheldrick, G. *Acta. Crystallogr. Sect. C. Struct. Chem*. **71** (1), 3 (2015).

Table S1. Crystal data and structure refinement for 16b.

**Crystal data**	
Chemical formula	$C_{21}H_{16}ClNO_5$
M_r	397.80
Crystal system, space group	Triclinic, $P\bar{1}$
Temperature (K)	150
a, *b*, *c* (Å)	7.5431 (3), 11.1550 (4), 11.6105 (4)
a, b, g (°)	86.5273 (16), 73.3161 (16), 72.5596 (15)
V (Å^3)	892.45 (6)
Z	2
Radiation type	Mo *K*a
m (mm^{-1})	0.25
Crystal shape	Block
Colour	Colourless
Crystal size (mm)	0.50 × 0.37 × 0.12
**Data collection**	
Diffractometer	Bruker AXS D8 Quest diffractometer with PhotonII charge-integrating pixel array detector (CPAD)
Radiation source	fine focus sealed tube X-ray source
Detector resolution (pixels mm^{-1})	7.4074
Absorption correction	Multi-scan *SADABS* 2016/2: Krause, L., Herbst-Irmer, R., Sheldrick G.M. & Stalke D. (2015). J. Appl. Cryst. 48, 3-10.

T_{min}, T_{max}	0.685, 0.746
No. of measured, independent and observed [$I > 2s(I)$] reflections	84259, 5960, 5372
R_{int}	0.036
(sin q/l)$_{max}$ (Å^{-1})	0.737
**Refinement**	
$R[F^2 > 2s(F^2)]$, $wR(F^2)$, S	0.032, 0.091, 1.04
No. of reflections	5960
No. of parameters	258
H-atom treatment	H atoms treated by a mixture of independent and constrained refinement
Dρ$_{max}$, Dρ$_{min}$ (e Å^{-3})	0.46, -0.26

Cells, viruses, and antiviral activity.

VeroE6 cells and TMPRSS2-overexpressing VeroE6 (VeroE6TMPRSS2) cells were obtained from the Japanese Collection of Research Bioresources (JCRB) Cell Bank (Osaka, Japan). VeroE6 cells were maintained in Dulbecco's modified Eagle's medium (d-MEM) supplemented with 10% fetal bovine serum (FCS), 100 µg/ml of penicillin, and 100 µg/ml of streptomycin. VeroE6TMPRSS2 cells were maintained in d-MEM as reported (ref.1) in the presence of 1 mg/ml of G418. SARS-CoV-2 strain JPN/TY/WK-521 (SARS-CoV-2WK-521) was obtained from the National Institute of Infectious Diseases (Tokyo, Japan).

Antiviral assay was carried out as described recently (reference 6): Cells were seeded in a 96-well plate ($2x10^4$ cells/well) and incubated. After 24 h, virus was inoculated into cells at multiplicity of infection (MOI) of 0.05. After an additional 72 h, cell culture supernatants were harvested and viral RNA was extracted using a QIAamp viral RNA minikit (Qiagen, Hilden, Germany), and quantitative RT-PCR (RT-qPCR) was then performed using One Step PrimeScript III RT-qPCR mix (TaKaRa Bio, Shiga, Japan) following the instructions of the manufacturers. The primers and probe used for detecting SARS-CoV-2 envelope (6) were 5=-ACT TCT TTT TCT TGC TTT CGT GGT-3= (forward), 5=-GCA GCA GTA CGC ACA CAA TC-3= (reverse), and 5=-FAM-CTA GTT ACA CTA GCC ATC CTT ACT GC-black hole quencher 1 (BHQ1)-3= (probe). To determine the cytotoxicity of each compound, cells were seeded in a 96-well plate (2_104 cells/well). One day later, various concentrations of each compound were added, and cells were incubated for additional 3 days. The 50% cytotoxic concentrations (CC50) values were determined using the WST-8 assay and Cell Counting Kit-8 (Dojindo, Kumamoto, Japan).

Antiviral activity of select compounds was also evaluated in VeroE6 cells in the context of P-glycoprotein efflux inhibitor CP-100356. The ability of the compounds to protect against SARS-CoV-2 induced cytopathic effect (CPE) or cell death was measured using a cell-viability based approach. Briefly, Vero E6 cells (American Type Culture Collection) seeded in 96 well plates were treated with varying

concentrations of test compounds or vehicle (0.53% DMSO) in the presence of 2μM CP-100356 (Axon Medchem LLC) in replicates of 6. Half of the replicate wells per compound dose (3 of 6) were challenged with SARS-CoV-2 virus at an MOI of 0.01 concurrent with the addition of compounds while the other wells (n=3) served as mock-uninfected controls. The plates were incubated for 72 hours and the viability of cells was assessed using CellTiter-Glo 2.0 Assay (Promega). The cytotoxicity of compounds was measured by comparing the viability of mock-uninfected cells treated with compounds relative to mock-uninfected vehicle control and CC_{50} was estimated. The antiviral efficacy was measured by comparing the viability of compound treated cells infected with virus relative to corresponding mock-uninfected cells and EC_{50} was estimated. Vehicle treated cells infected with SARS-CoV-2 show a viability of ~40% under these assay conditions. Increase in percent cellular viability ($40\% < x \leq 100\%$) with compound treatment therefore reflects inhibition of SARS-CoV-2. The experiments with live virus were performed in a Biosafety Level 3 laboratory at Purdue University using protocols approved by the Institutional Biosafety Committee. The infectious RNA for SARS-CoV-2/human/USA/WA-CDC-WA1/2020 (GenBank Accession: MN985325) was kindly provided by Prof. Michael S. Diamond (Washington University School of Medicine in St. Louis).

25. Hattori, S.-i.; Higshi-Kuwata, N.; Raghavaiah, J.; Das, D.; Bulut, H.; Davis, D.A.; Takamatsu, Y.; Matsuda, K.; Takamune, N.; Kishimoto, N.; et al. GRL-0920, an indole chloropyridinyl ester, completely blocks SARS-CoV-2 infection. *MBio* **2020**, *11*, e01833-20.

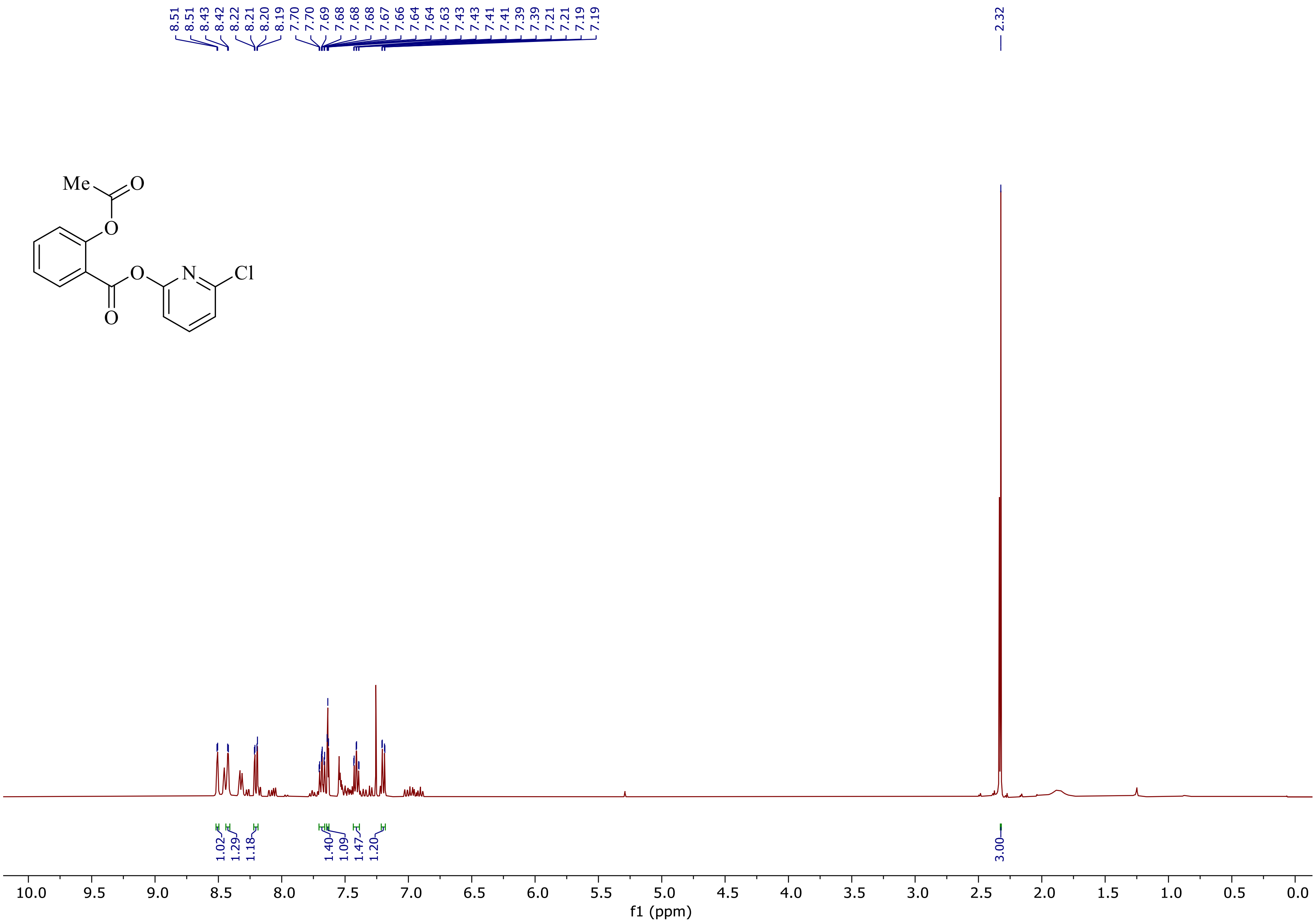

^{1}H NMR (400 MHz, $CDCl_3$) of Compound 6a

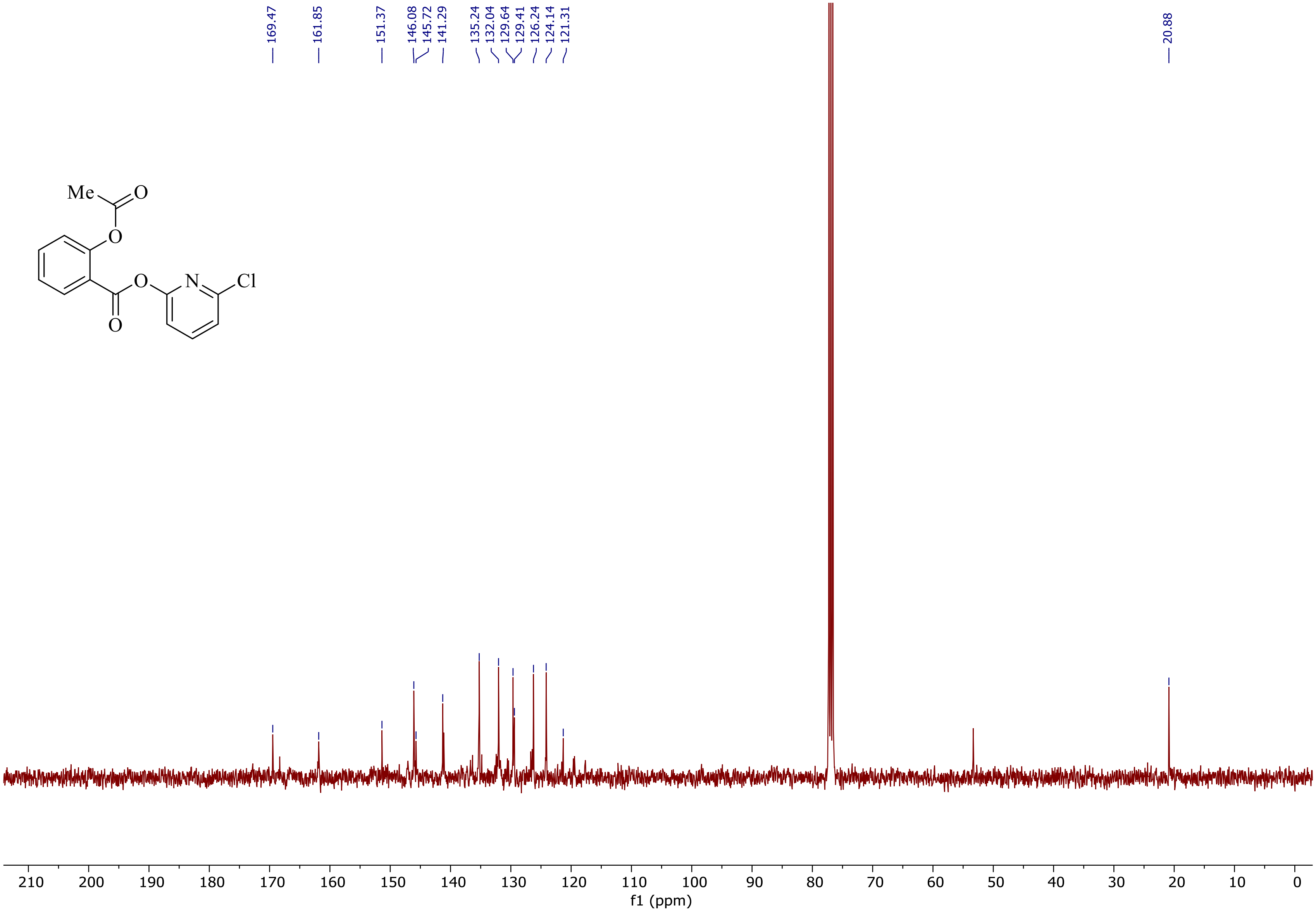

^{13}C NMR (100 MHz, CDCl$_3$) of Compound 6a

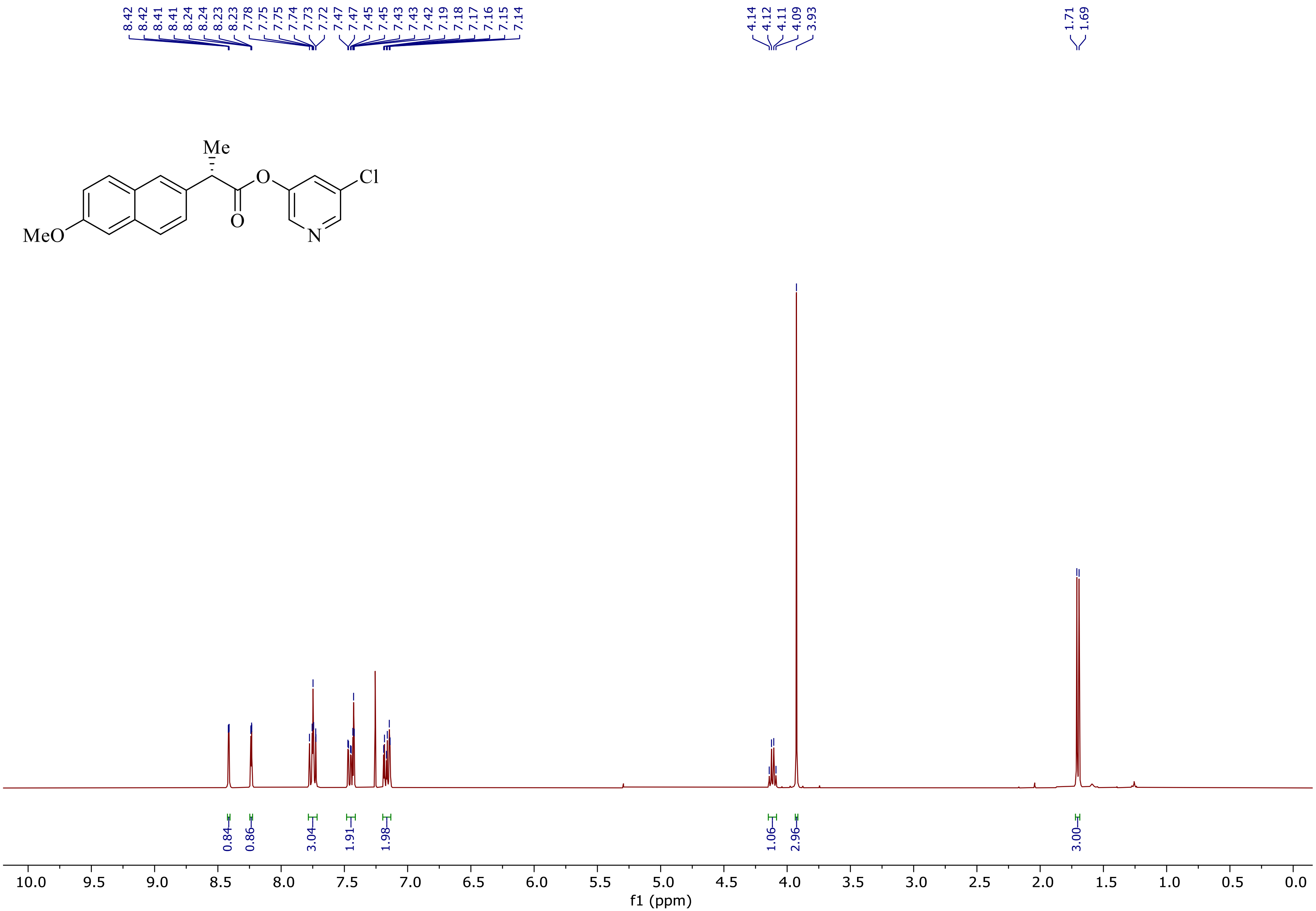

^{1}H NMR (400 MHz, $CDCl_3$) of Compound 8a

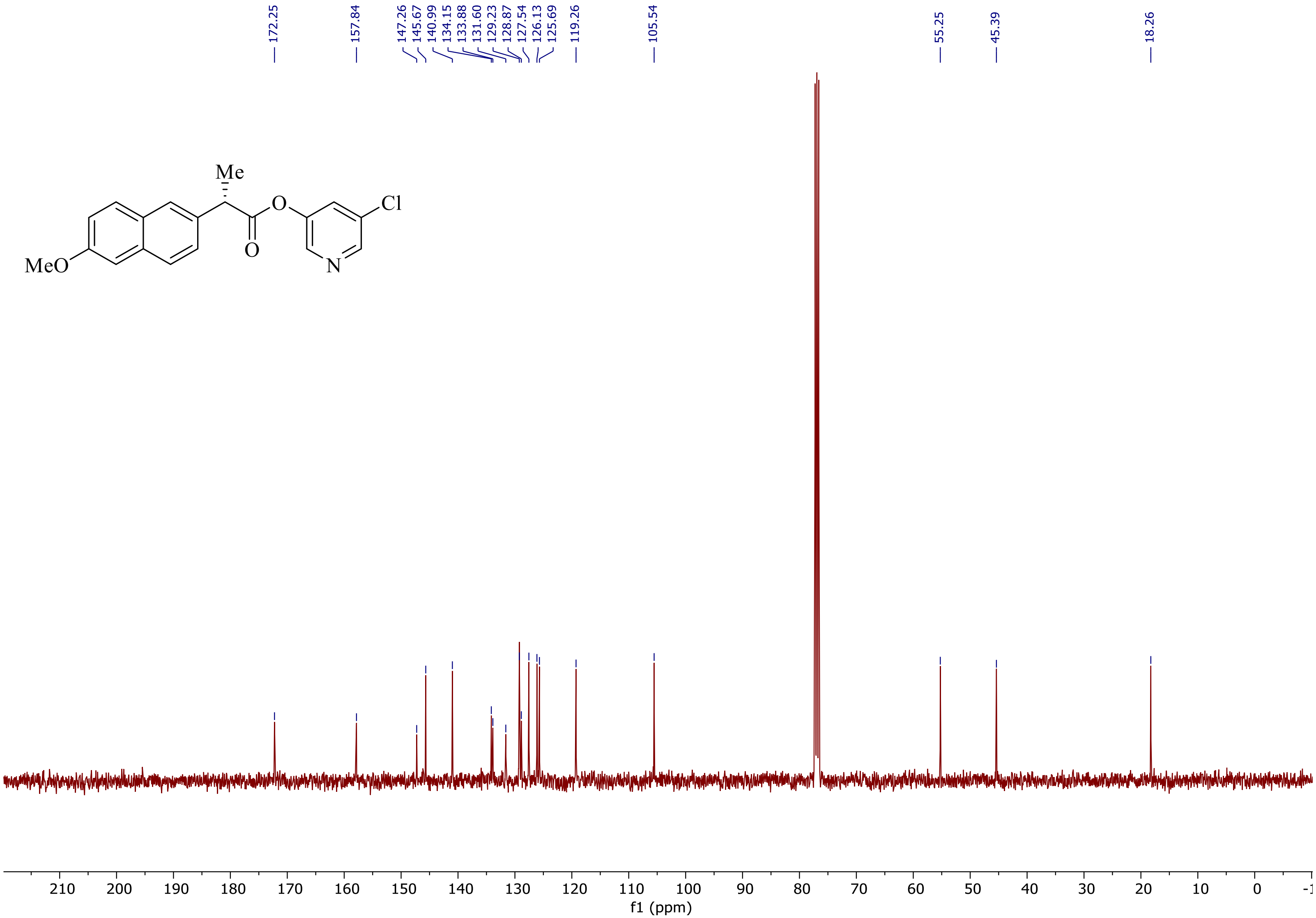

^{13}C NMR (100 MHz, $CDCl_3$) of Compound 8a

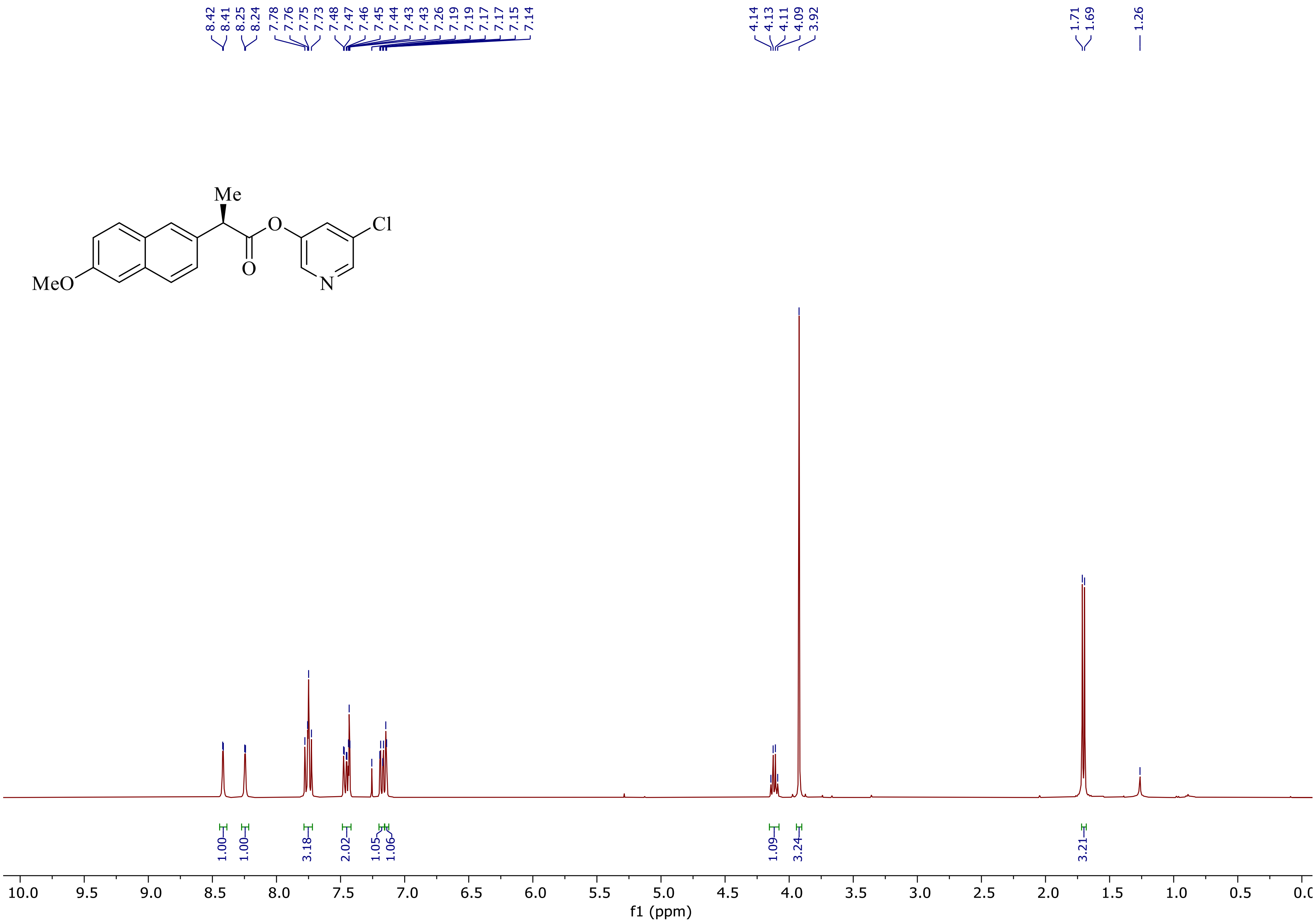

^{1}H NMR (400 MHz, $CDCl_3$) of Compound 9a

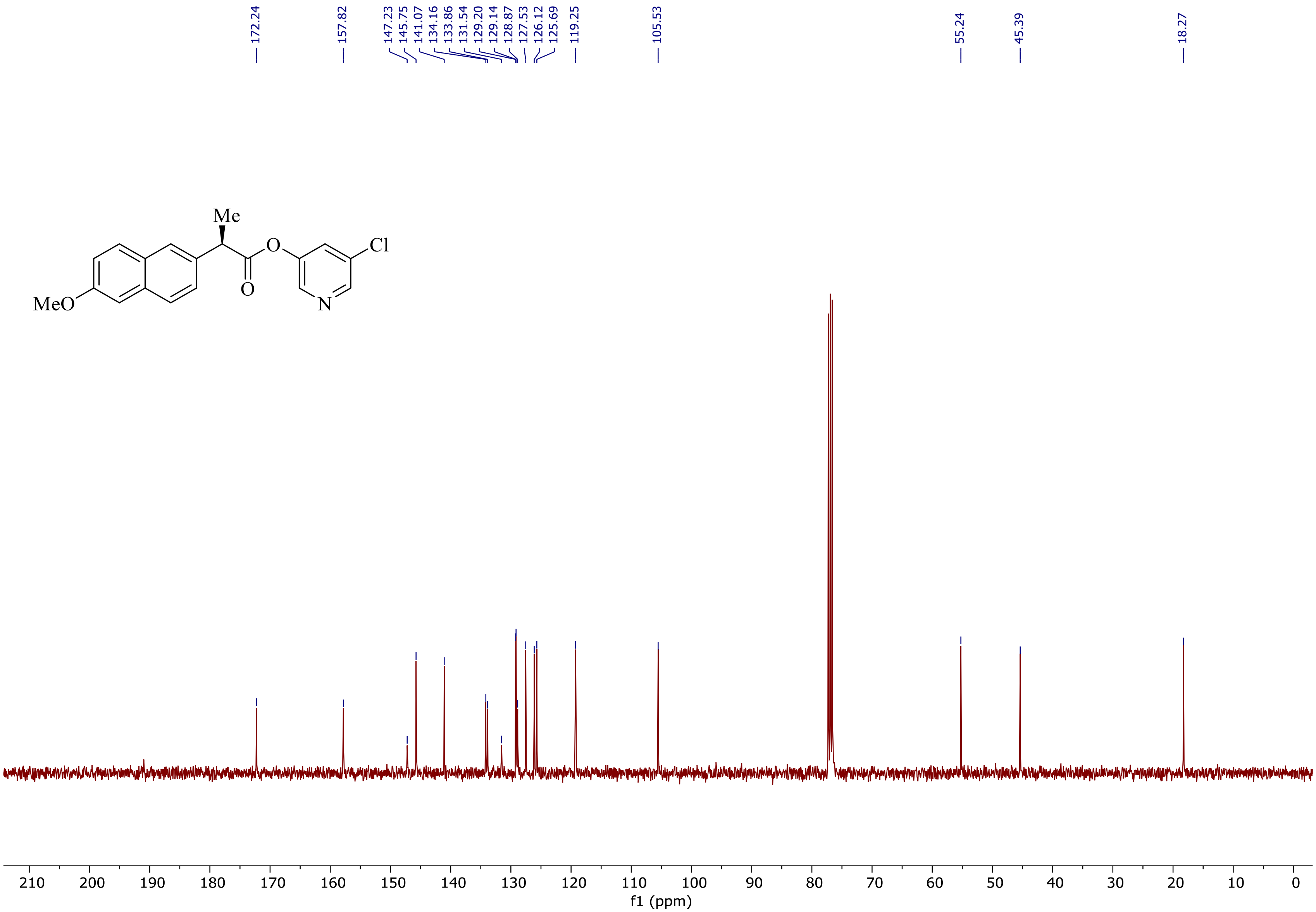

^{13}C NMR (100 MHz, $CDCl_3$) of Compound 9a

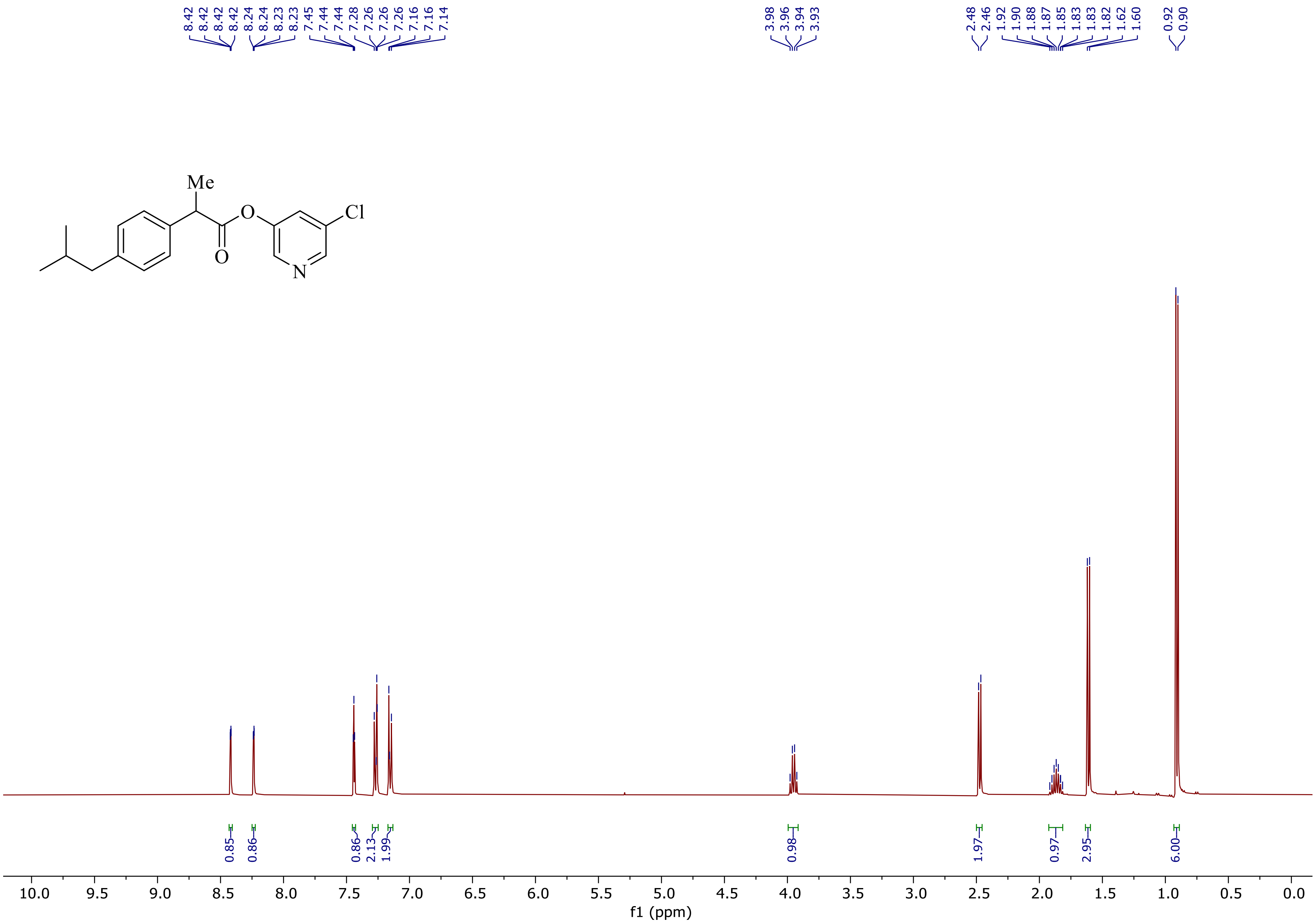

^{1}H NMR (400 MHz, $CDCl_3$) of Compound 10a

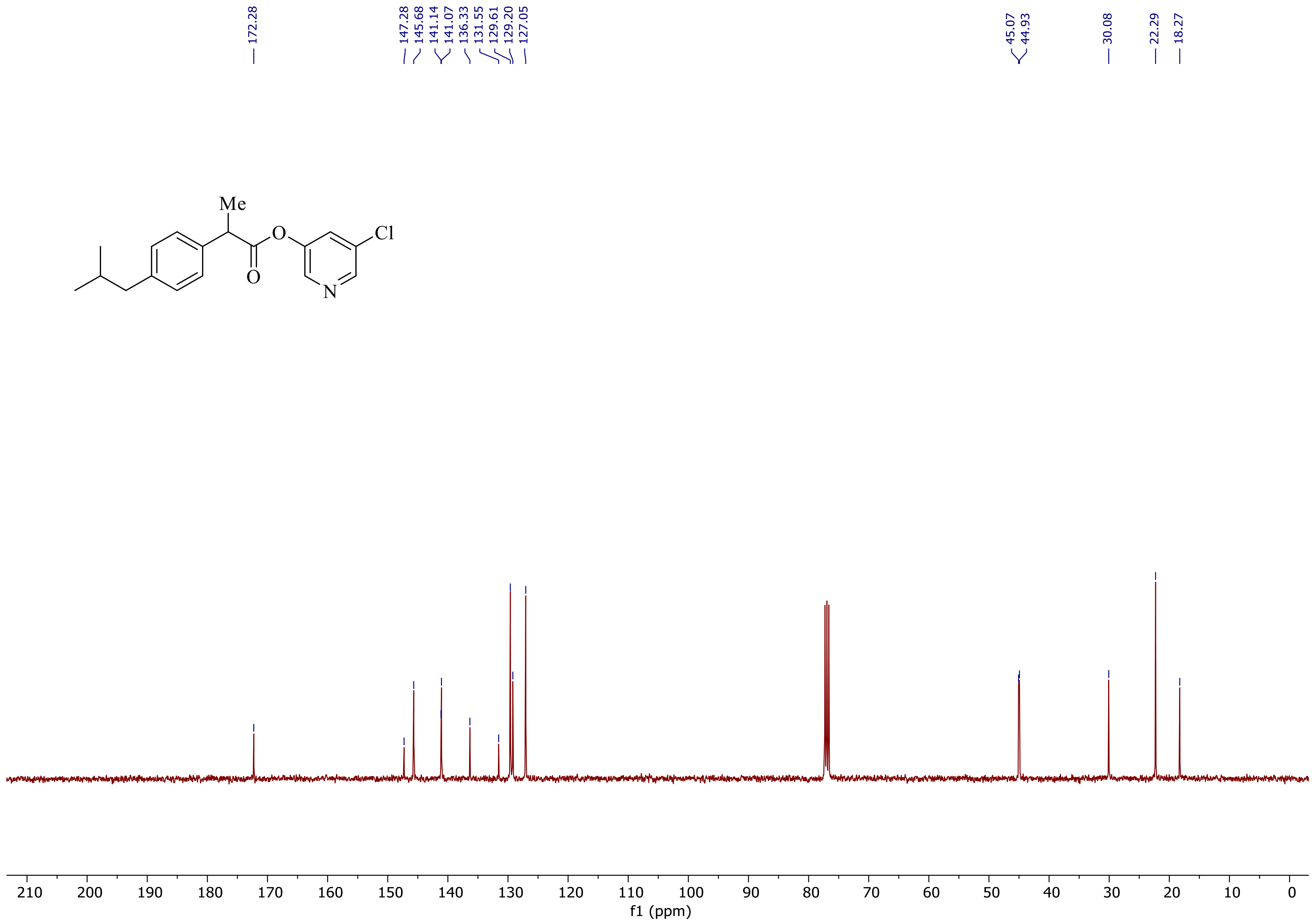
172.28
147.28
145.68
141.14
141.07
136.33
131.55
129.61
129.20
127.05
45.07
44.93
30.08
22.29
18.27
Me
O
Cl
O
N
210 200 190 180 170 160 150 140 130 120 110 100 90 80 70 60 50 40 30 20 10 0
f1 (ppm)

^{13}C NMR (100 MHz, $CDCl_3$) of Compound 10a

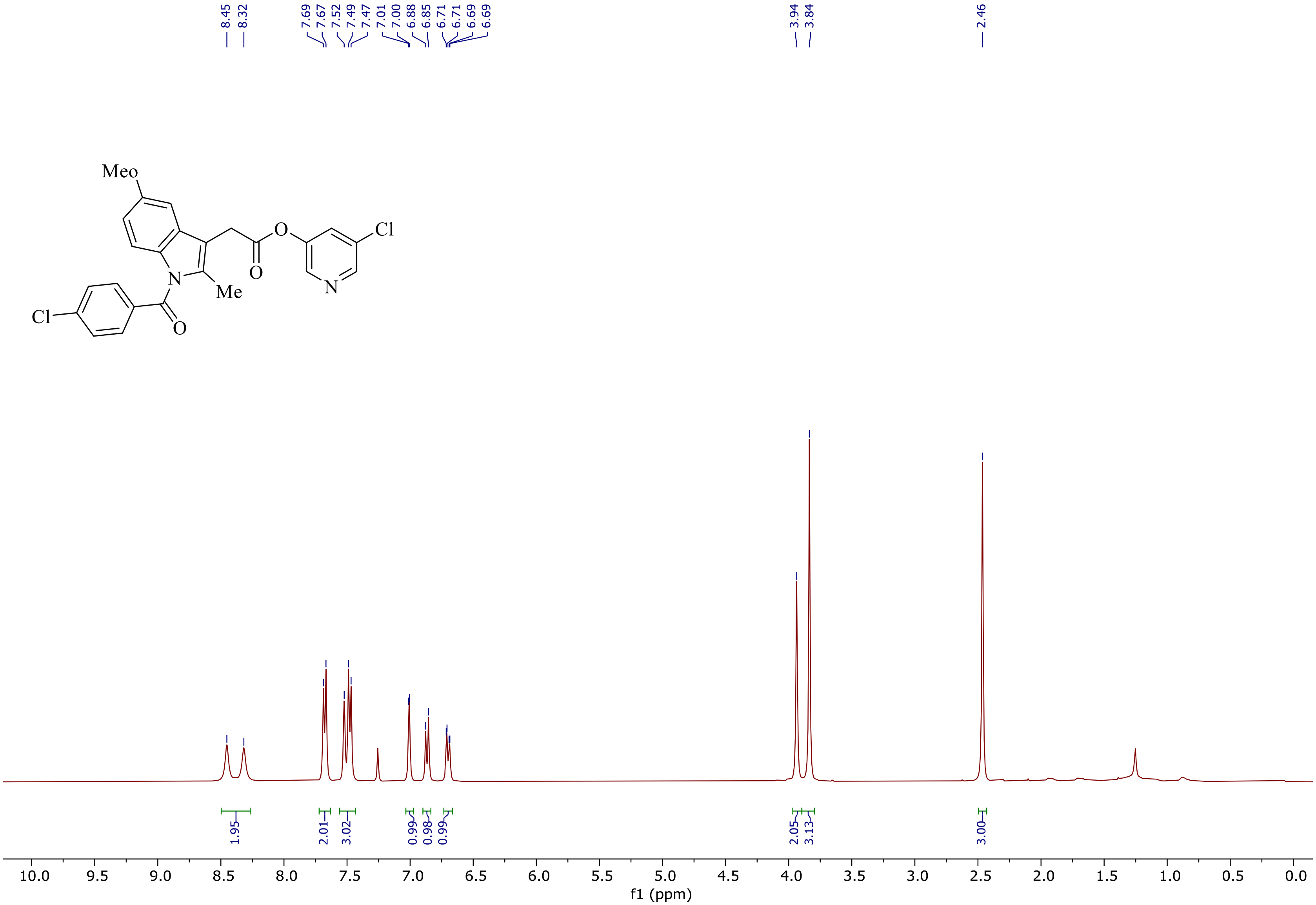

^{1}H NMR (400 MHz, $CDCl_3$) of Compound 11a

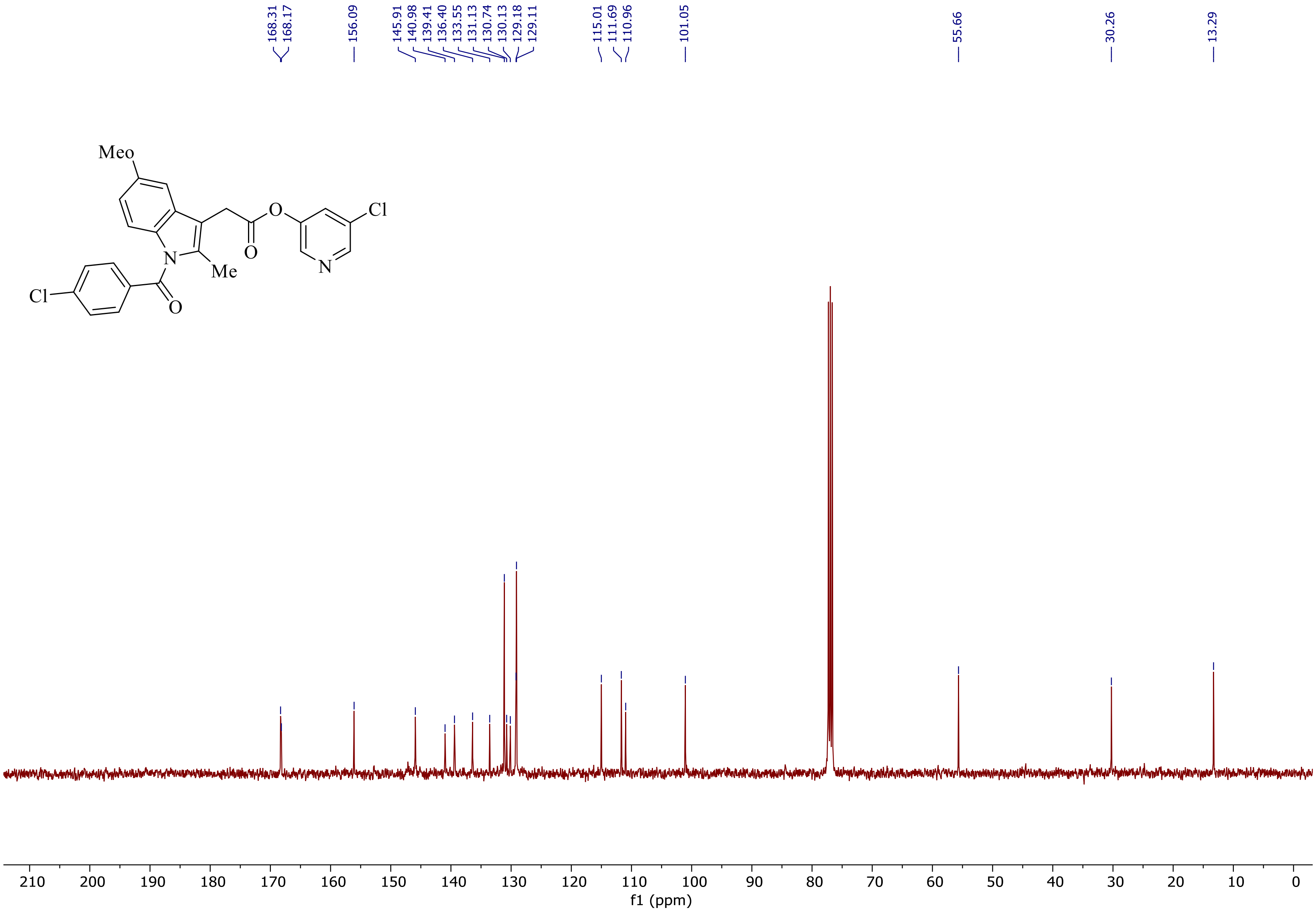

^{13}C NMR (100 MHz, $CDCl_3$) of Compound 11a

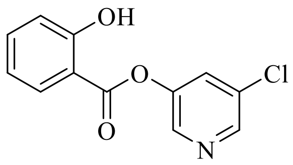

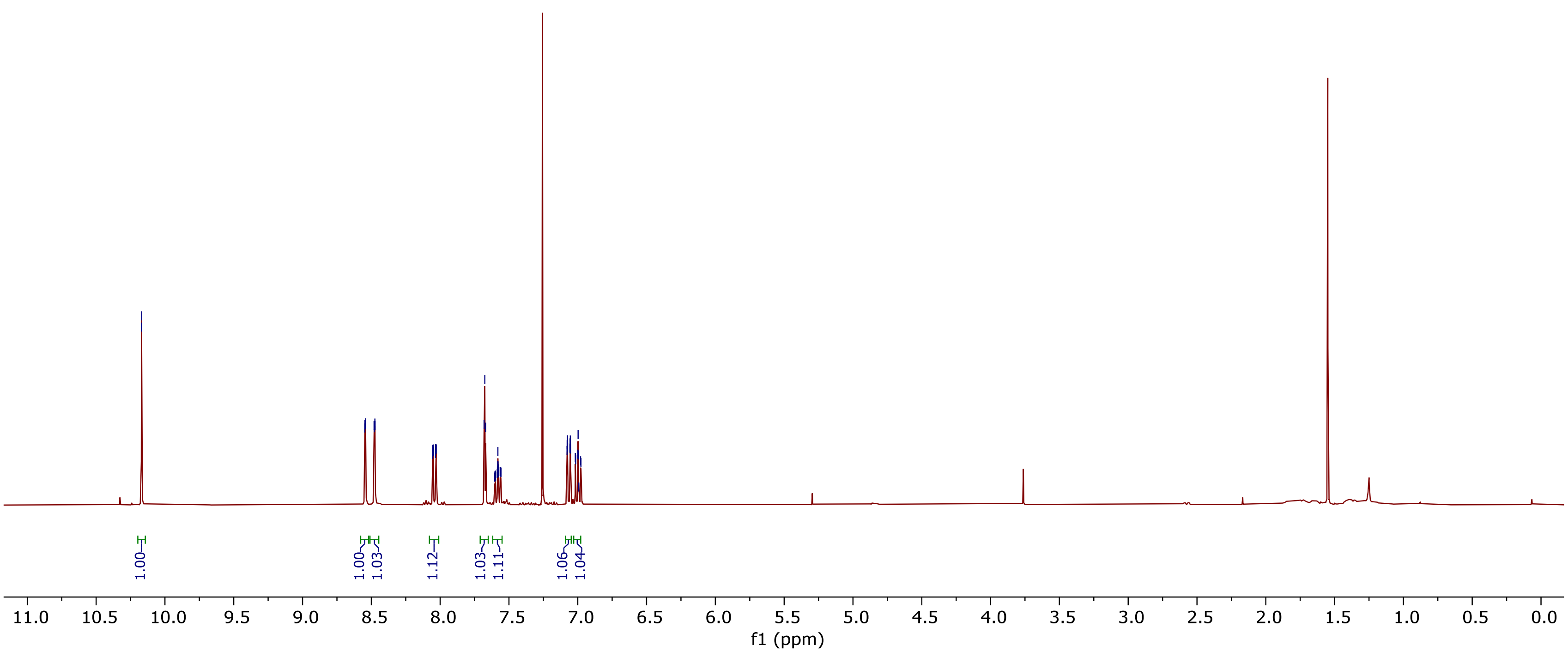

^{1}H NMR (400 MHz, $CDCl_3$) of Compound 12a

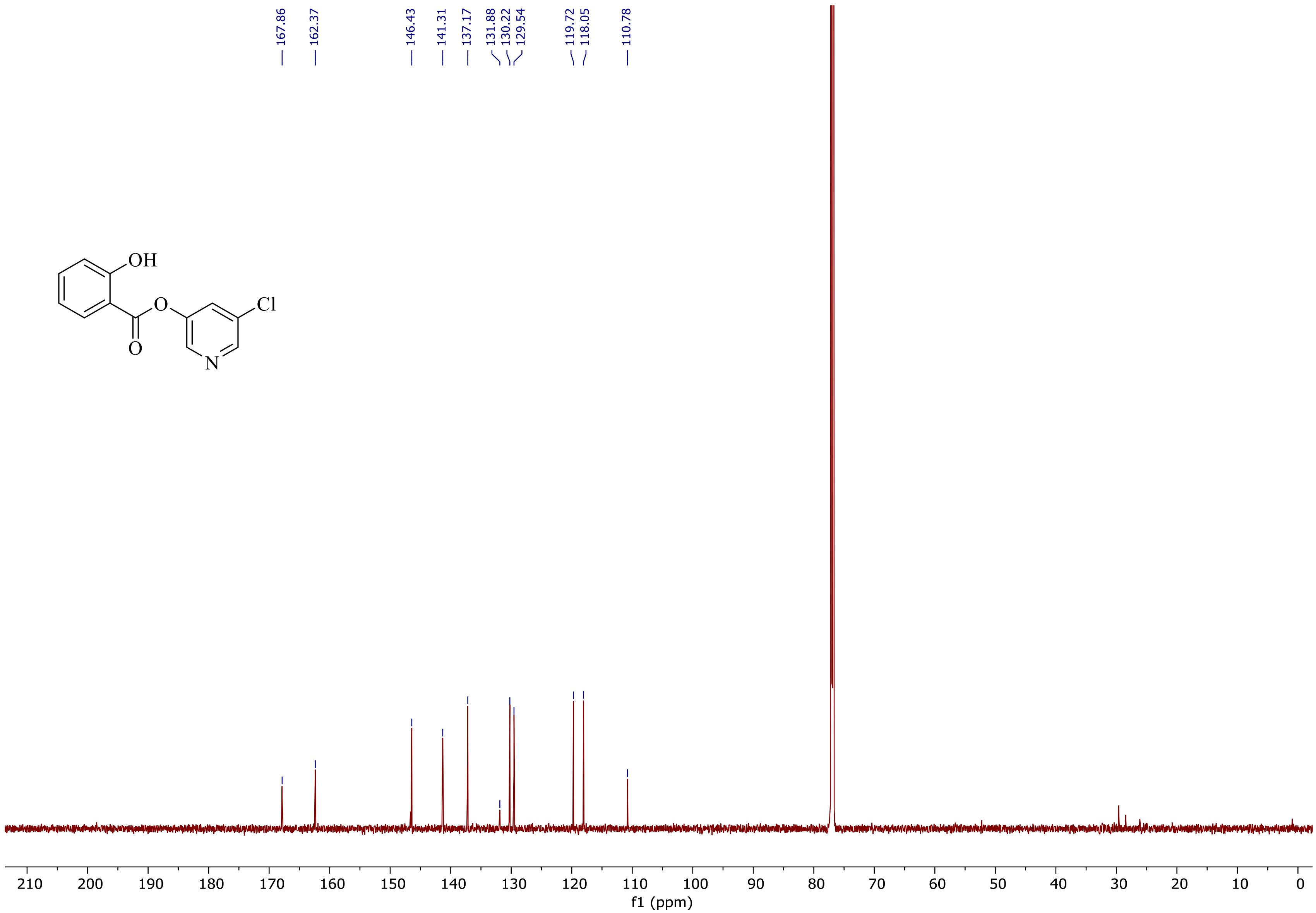

^{13}C NMR (100 MHz, $CDCl_3$) of Compound 12a

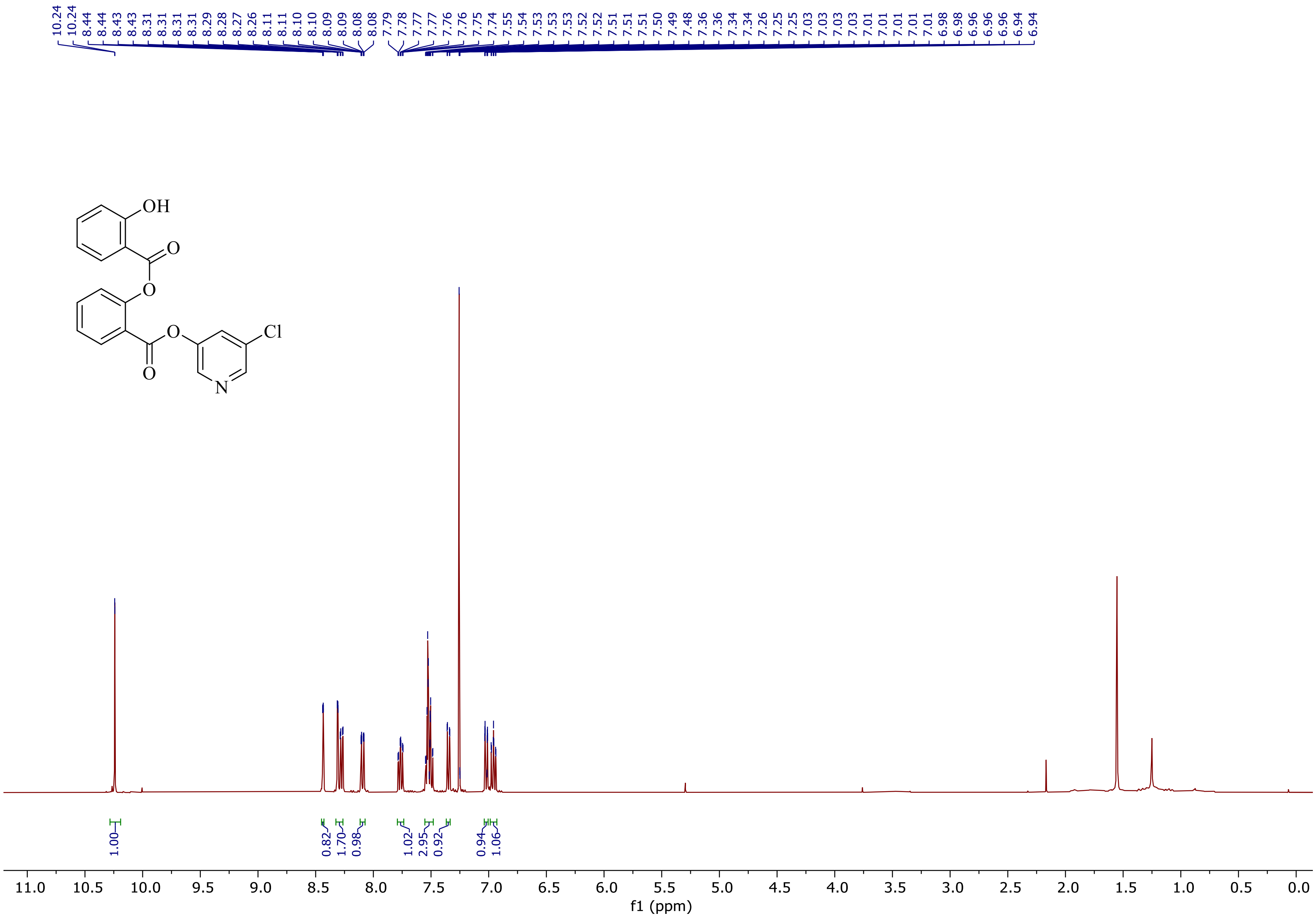

^{1}H NMR (400 MHz, $CDCl_3$) of Compound 12b

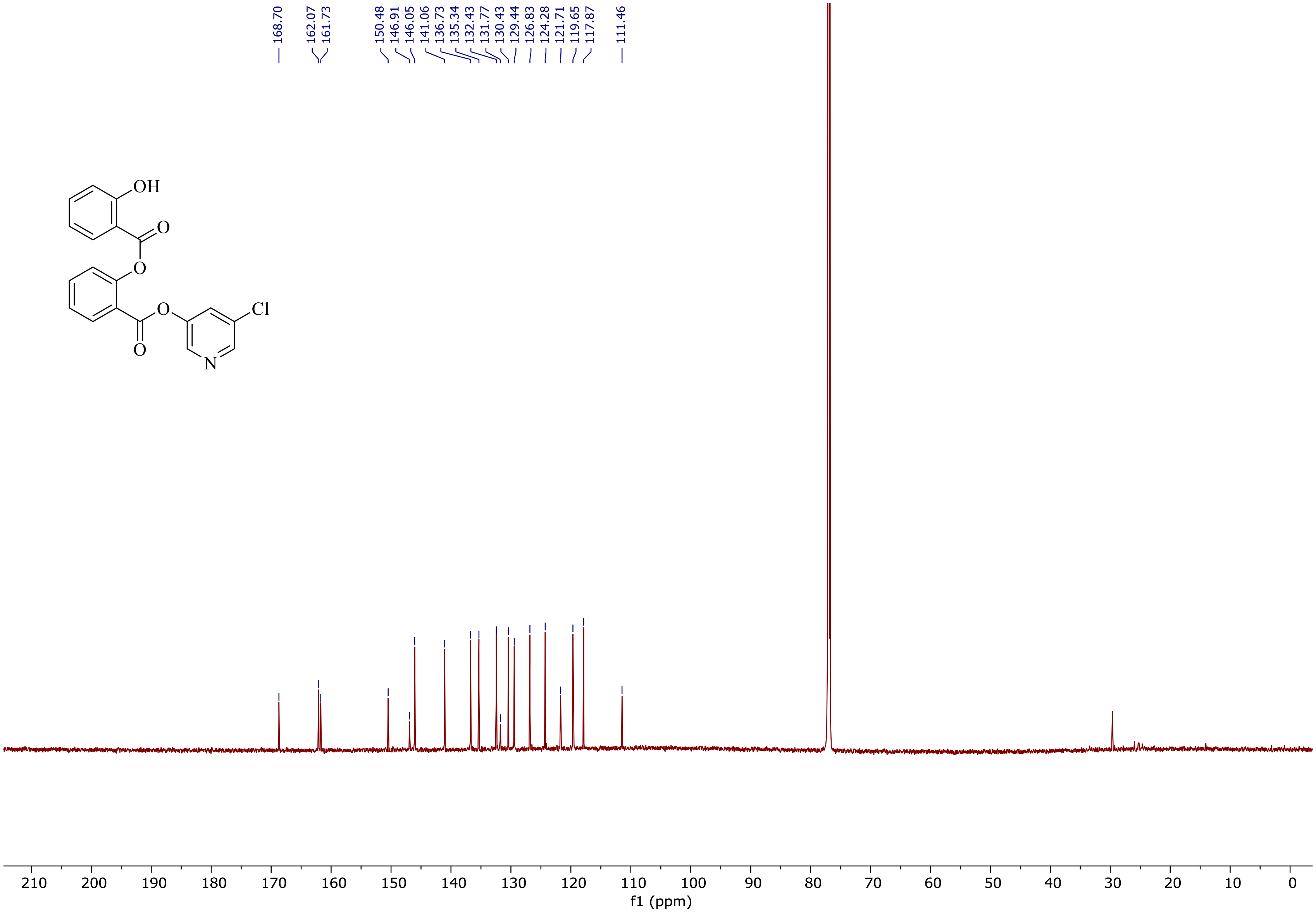

^{13}C NMR (200 MHz, $CDCl_3$) of Compound 12b

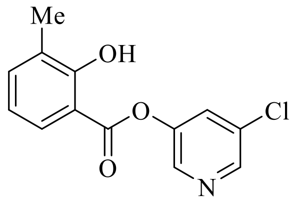

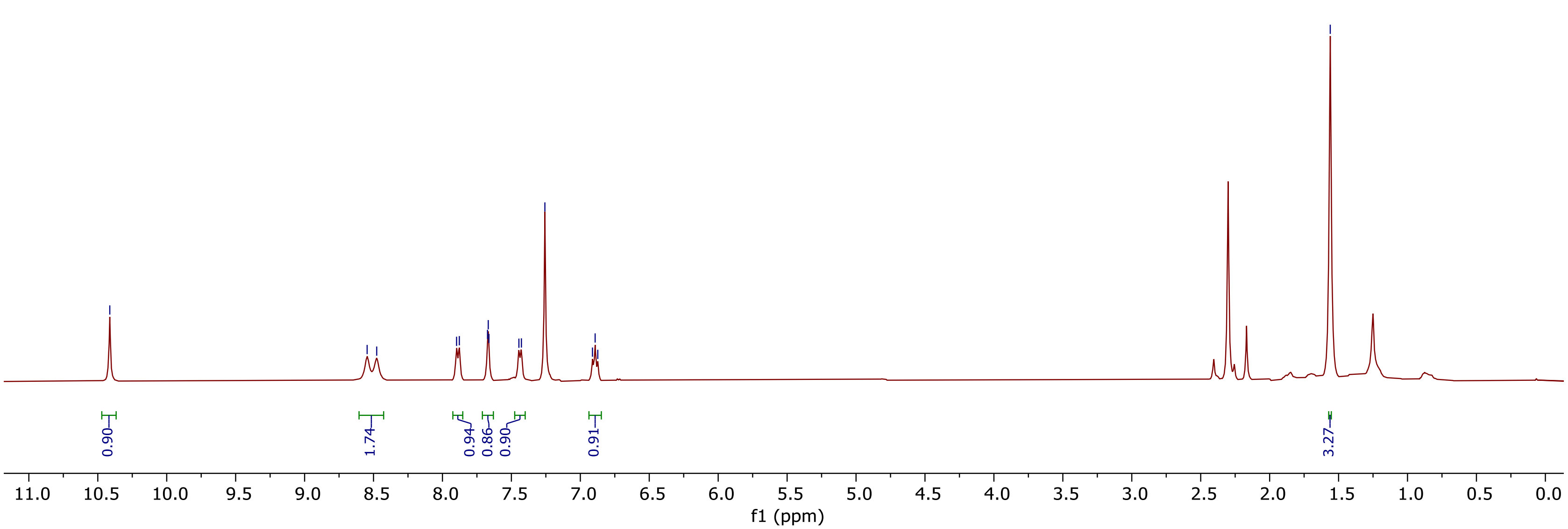

^{1}H NMR (400 MHz, $CDCl_3$) of Compound 13a

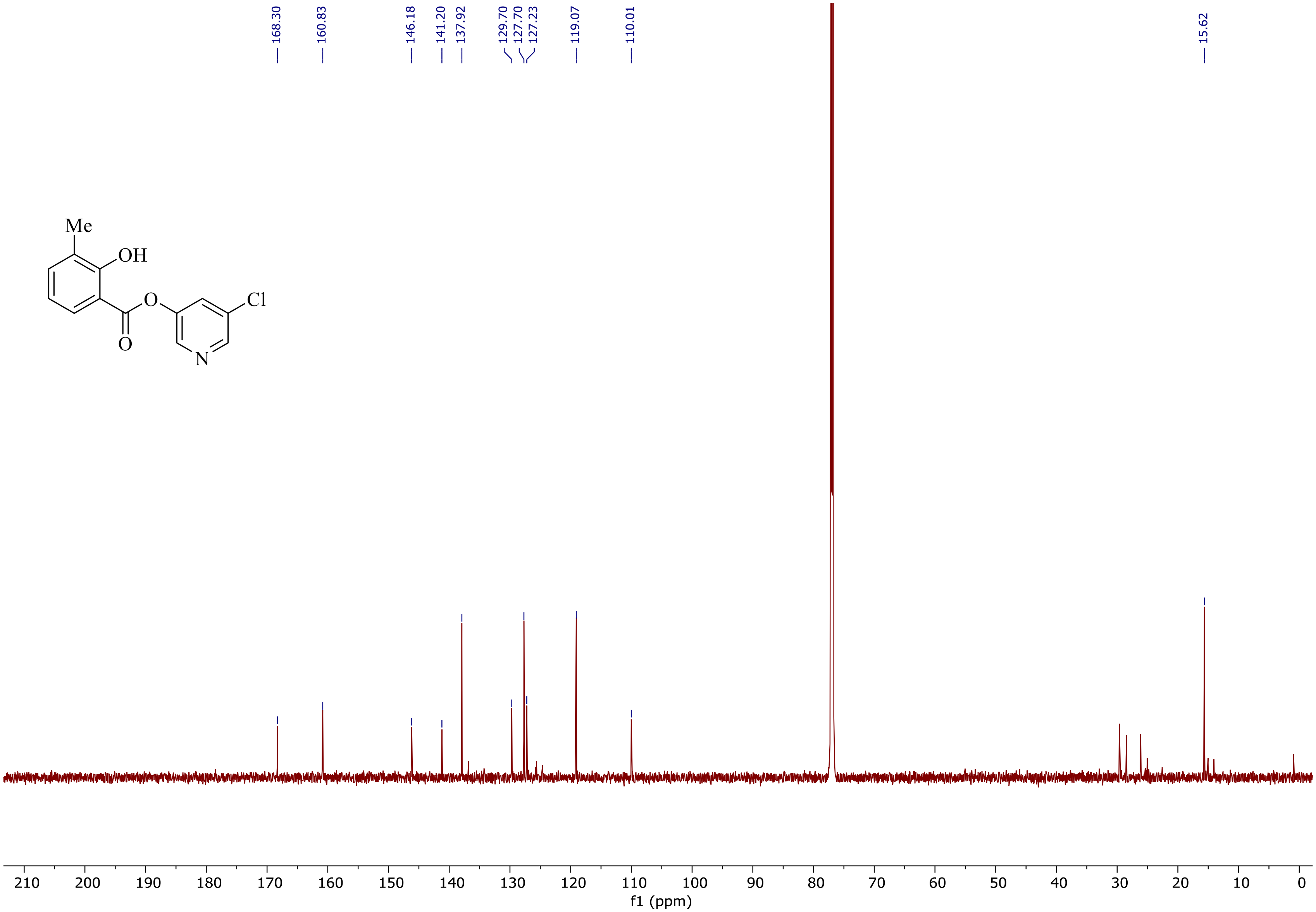

^{13}C NMR (200 MHz, $CDCl_3$) of Compound 13a

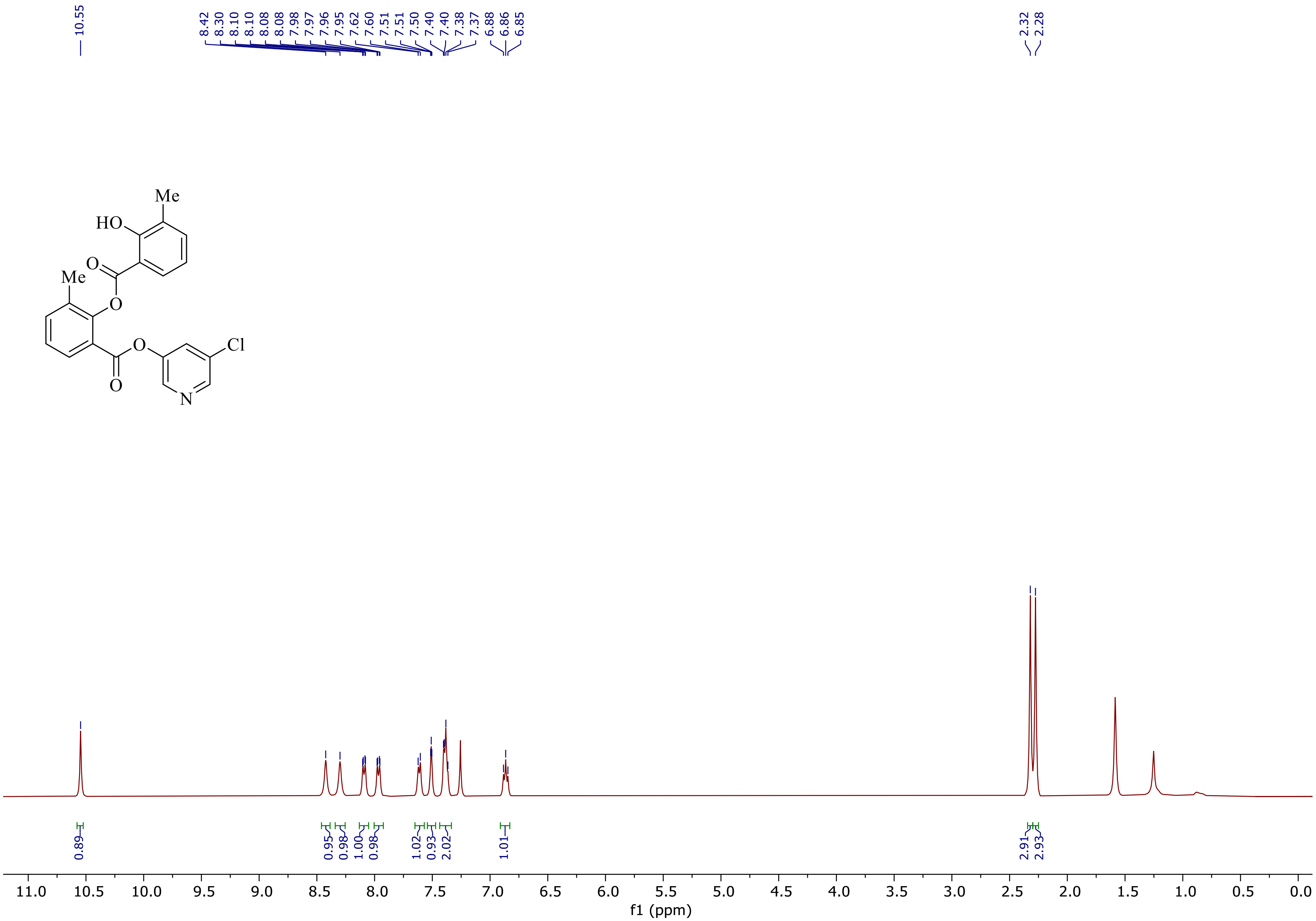

^{1}H NMR (400 MHz, $CDCl_3$) of Compound 13b

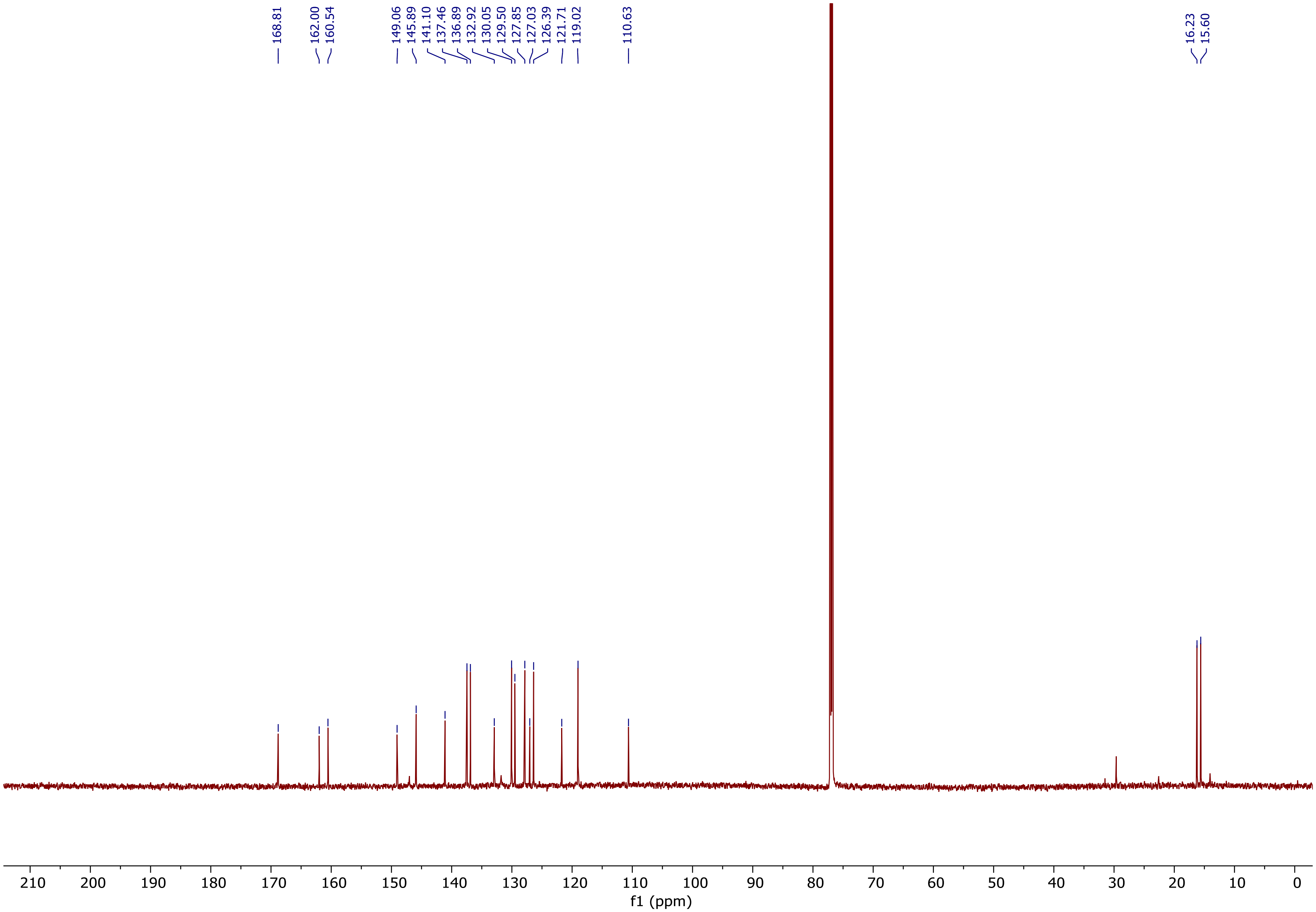

^{13}C NMR (200 MHz, $CDCl_3$) of Compound 13b

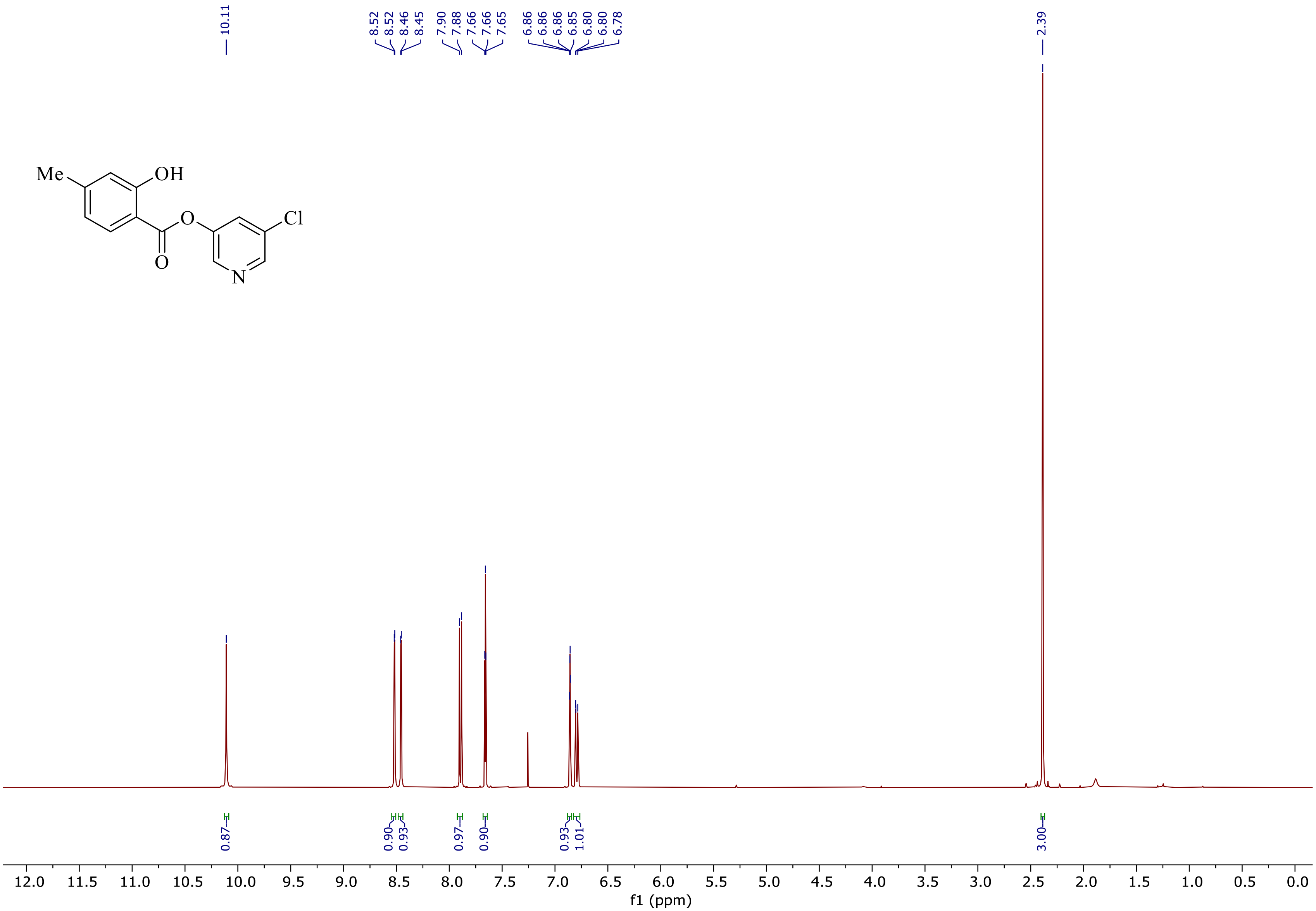

^{1}H NMR (400 MHz, $CDCl_3$) of Compound 14a

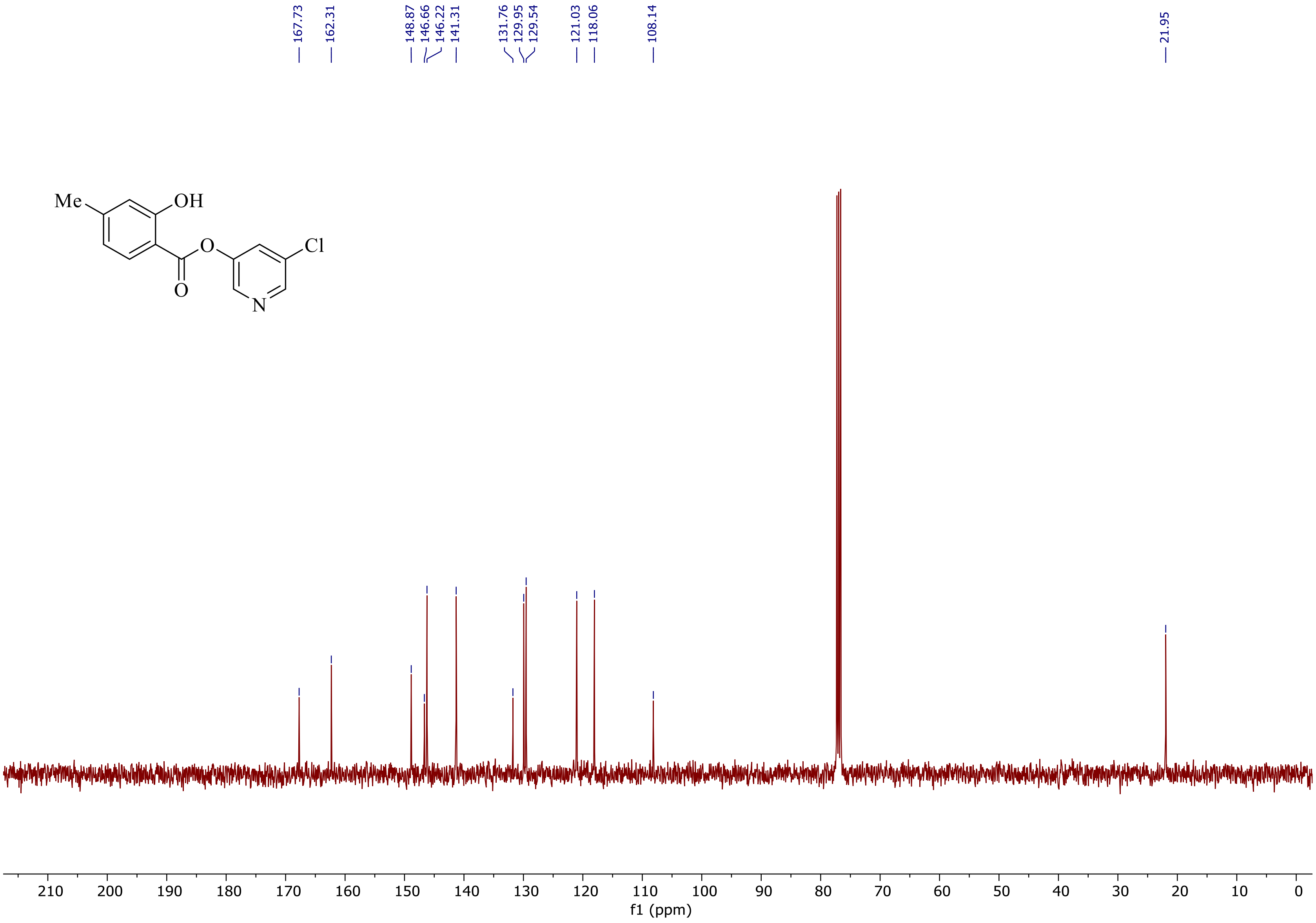

^{13}C NMR (100 MHz, $CDCl_3$) of Compound 14a

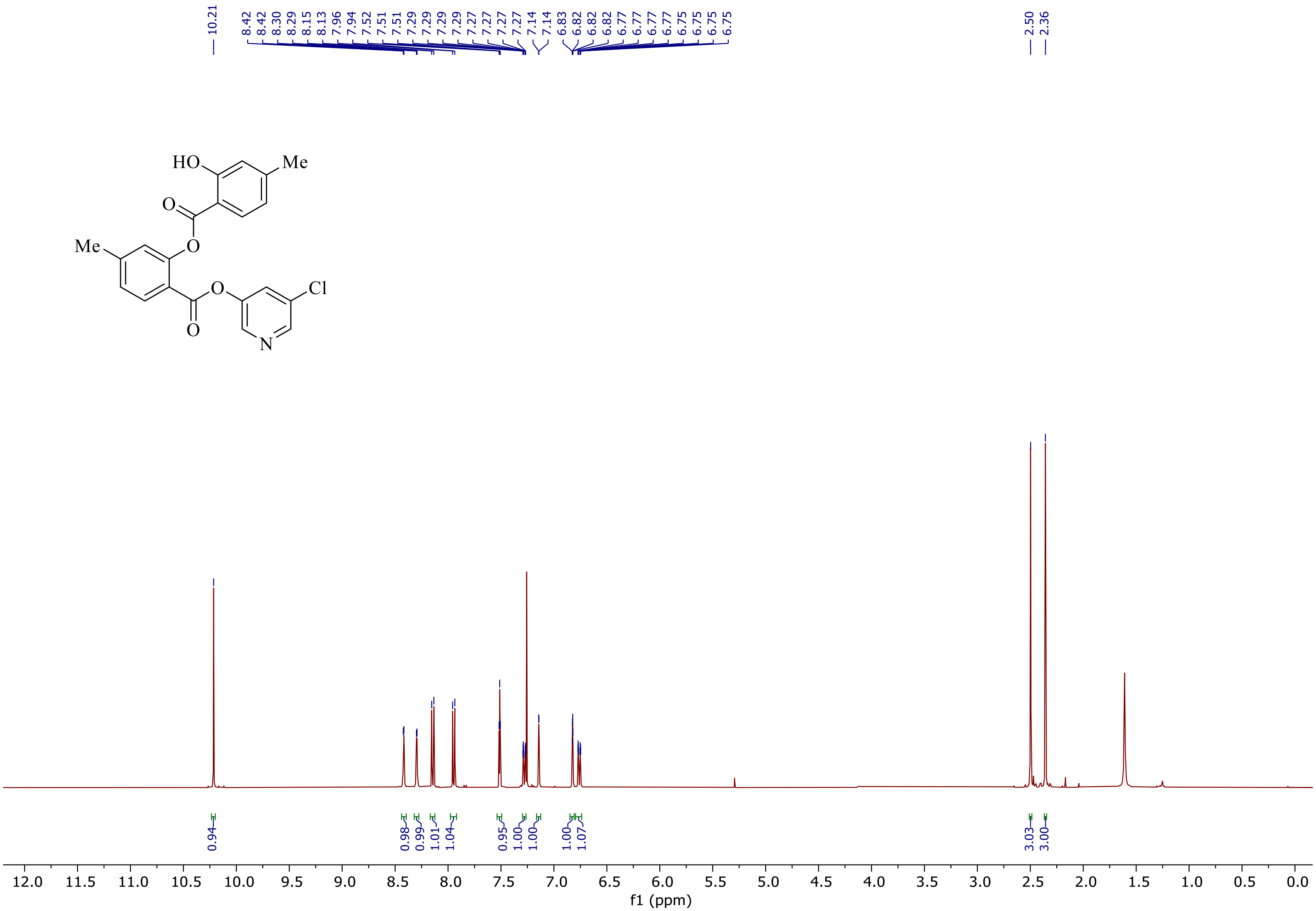

^{1}H NMR (400 MHz, $CDCl_3$) of Compound 14b

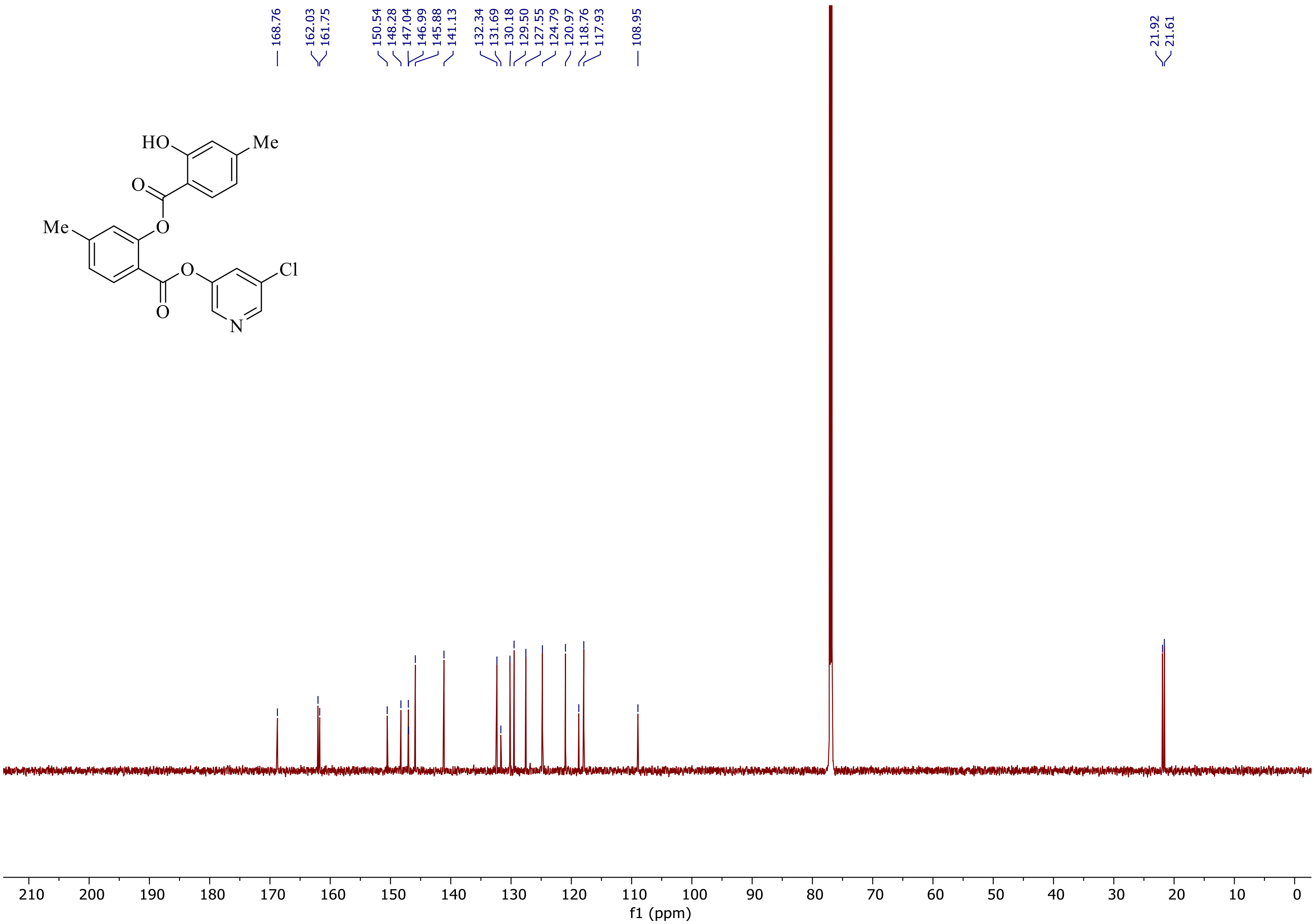

^{13}C NMR (200 MHz, $CDCl_3$) of Compound 14b

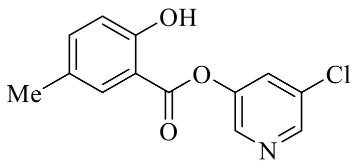

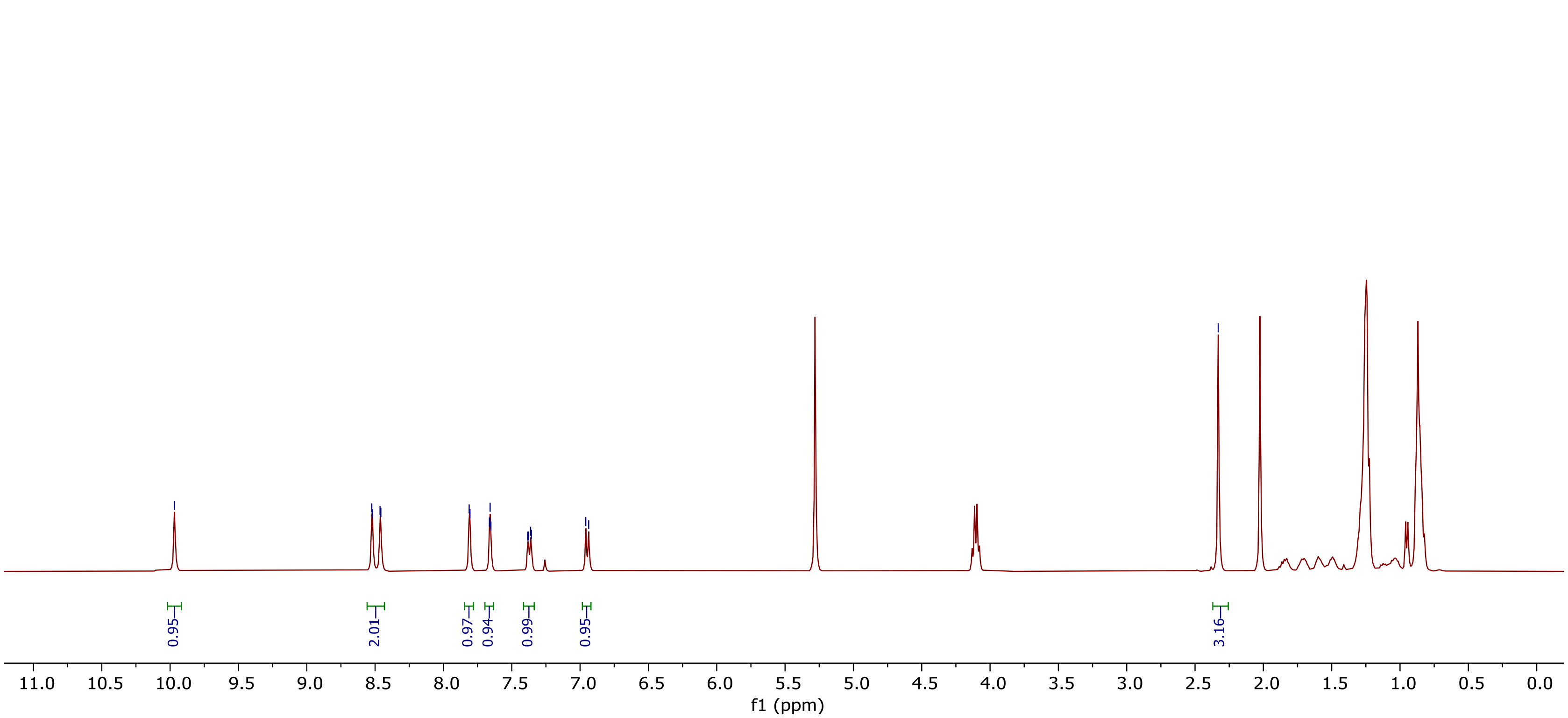

^{1}H NMR (400 MHz, CDCl$_3$) of Compound 15a

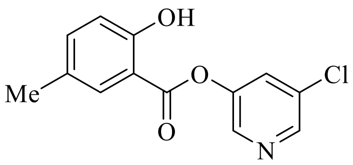

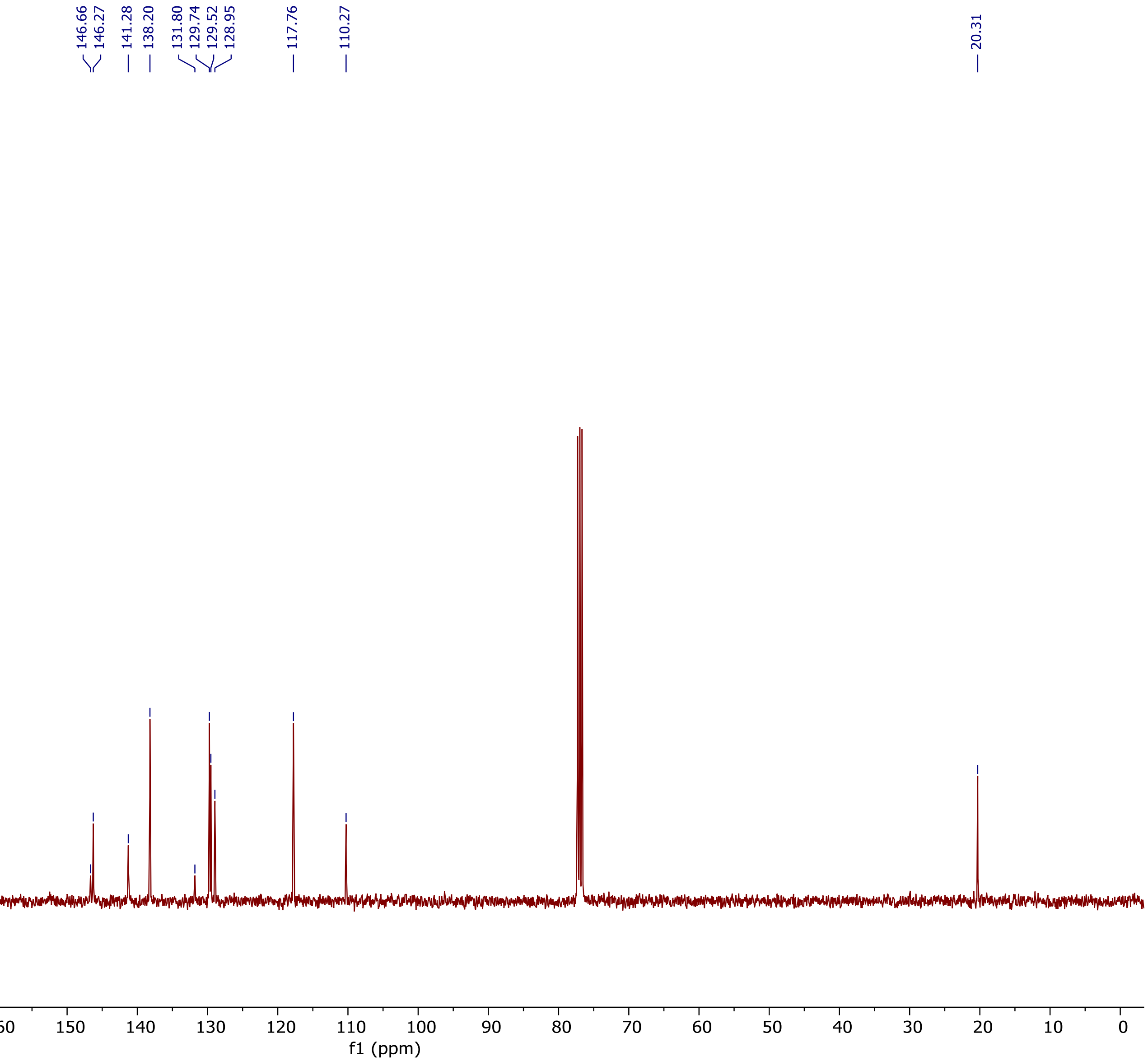

^{13}C NMR (100 MHz, $CDCl_3$) of Compound 15a

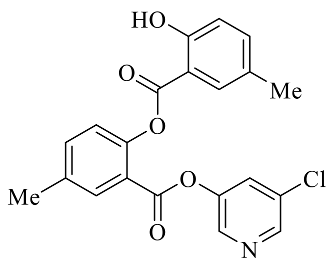

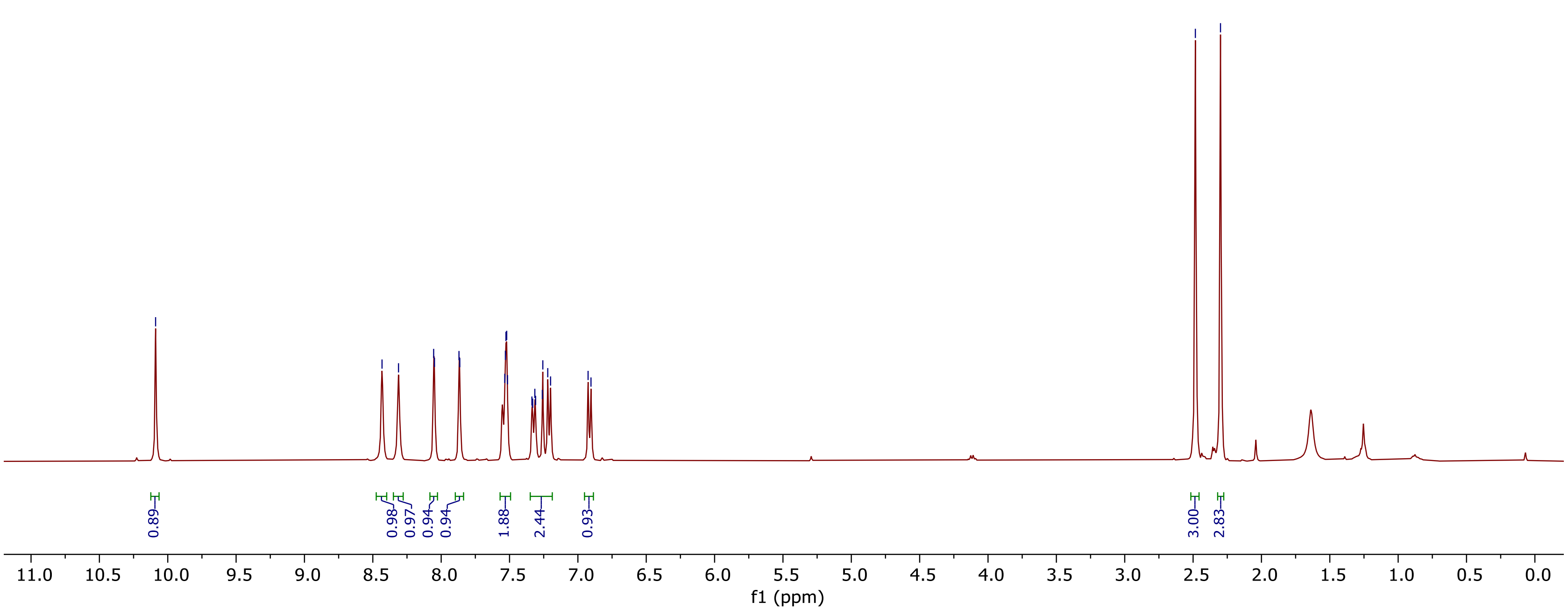

^{1}H NMR (400 MHz, CDCl3) of Compound 15b

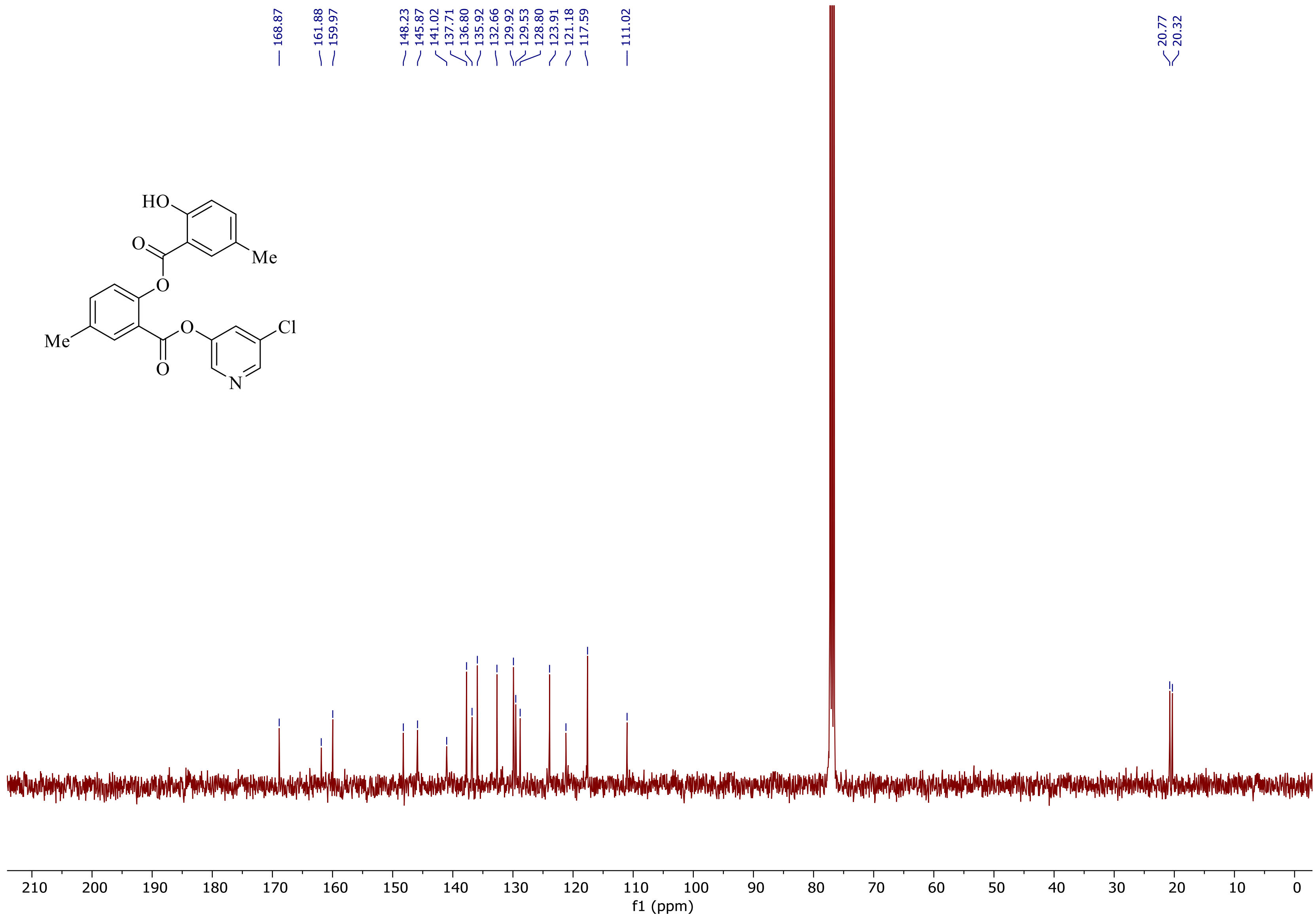

^{13}C NMR (100 MHz, $CDCl_3$) of Compound 15b

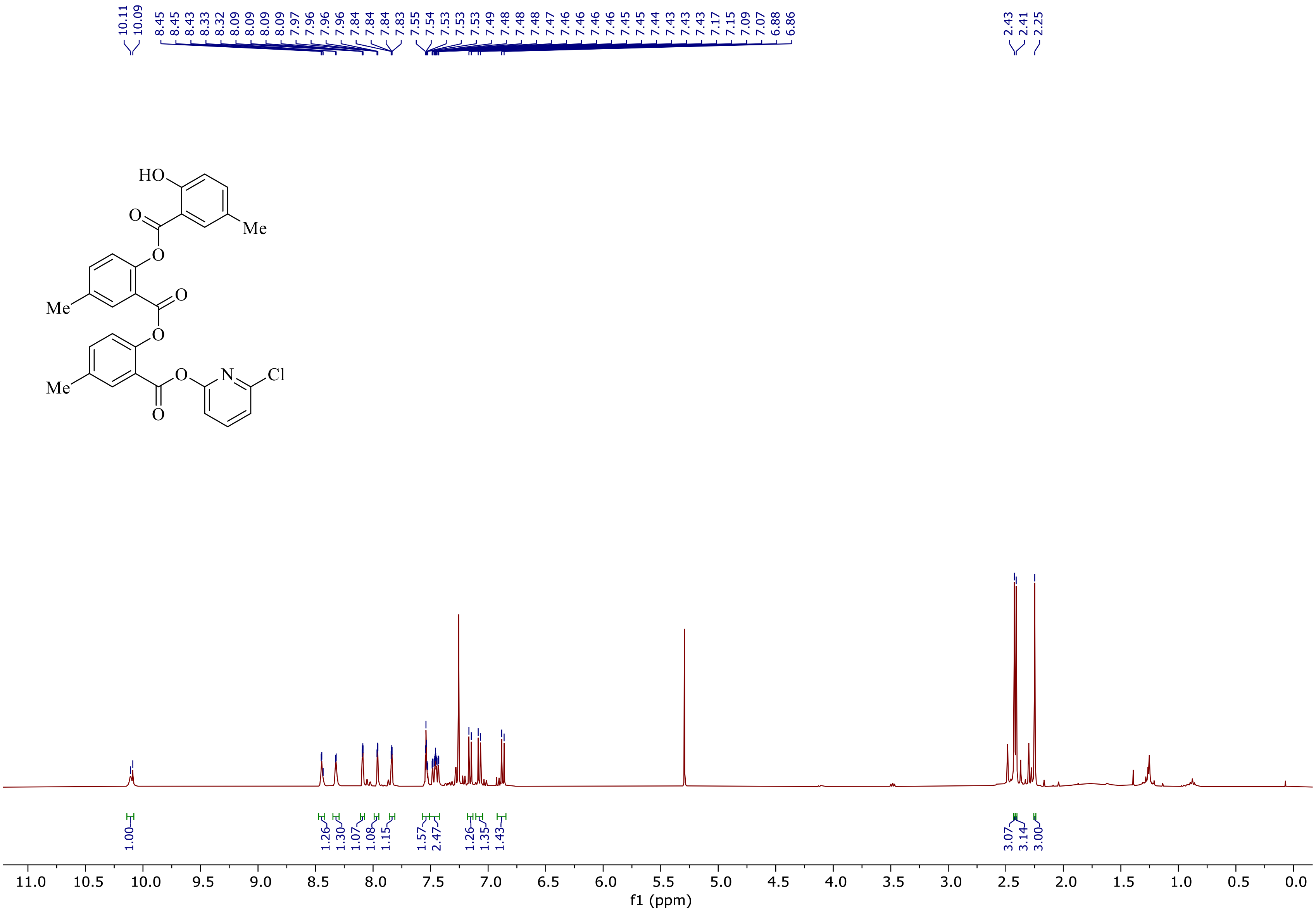

^{1}H NMR (400 MHz, $CDCl_3$) of Compound 15c

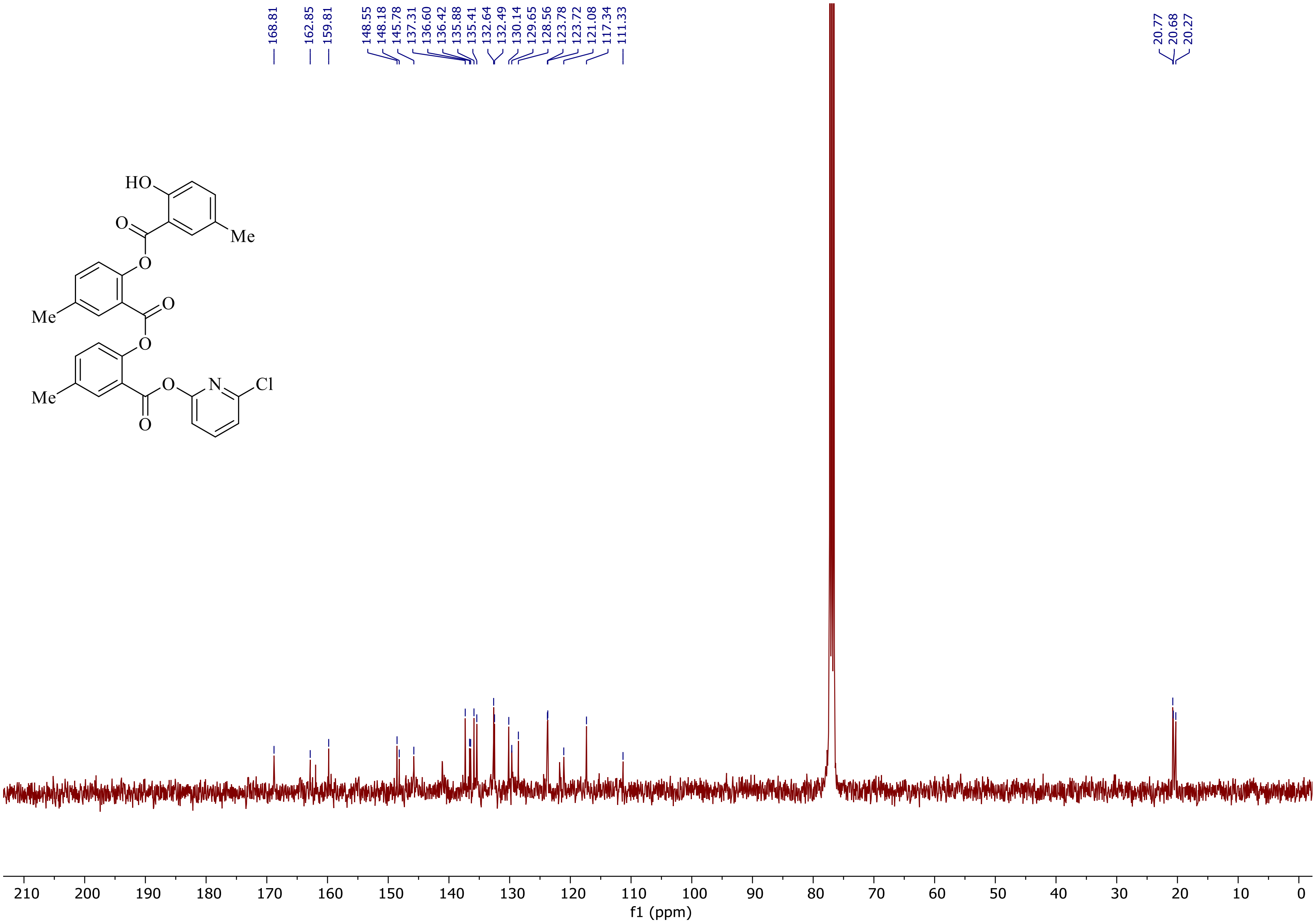

^{13}C NMR (100 MHz, CDCl$_3$) of Compound 15c

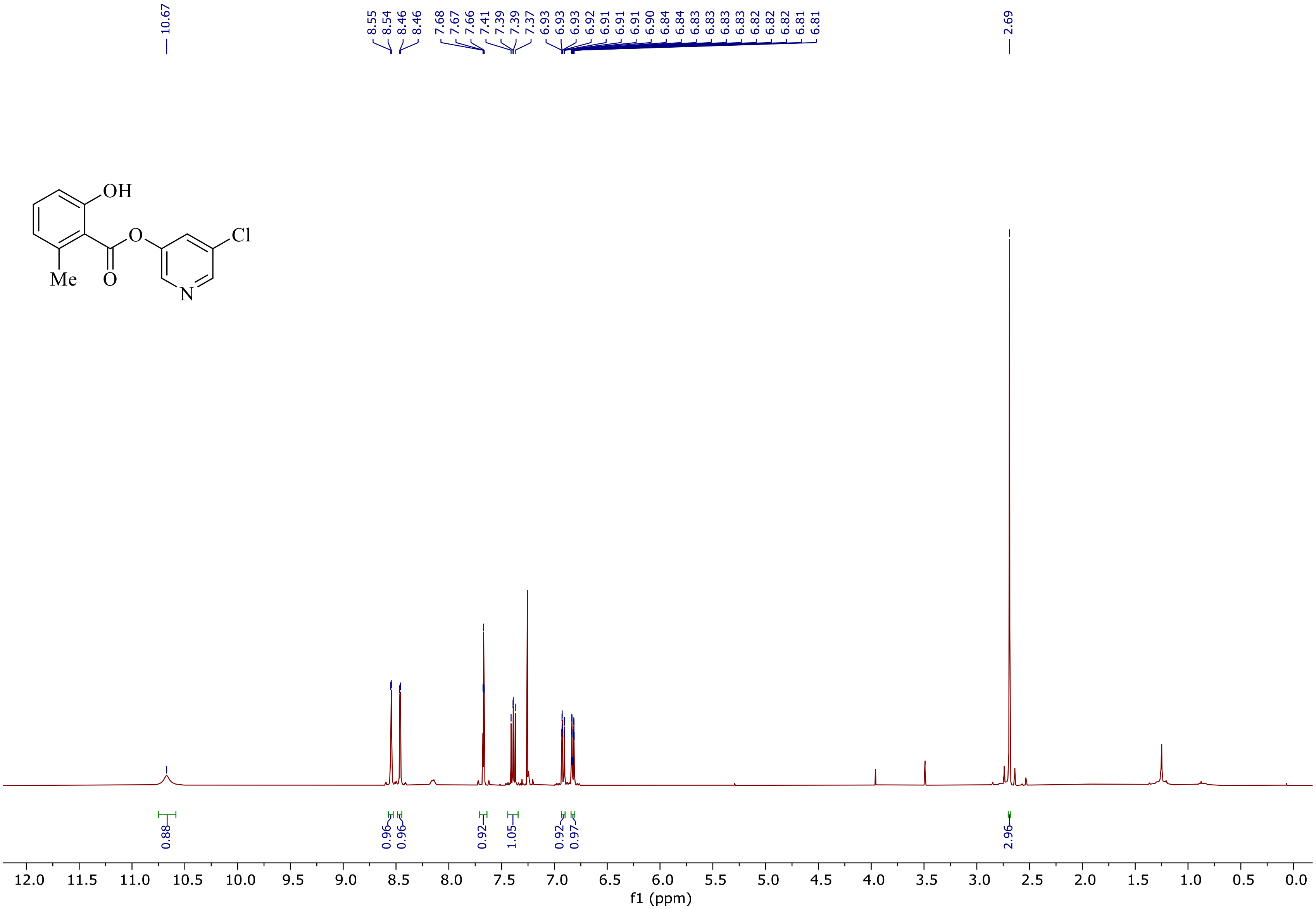

^{1}H NMR (400 MHz, $CDCl_3$) of Compound 16a

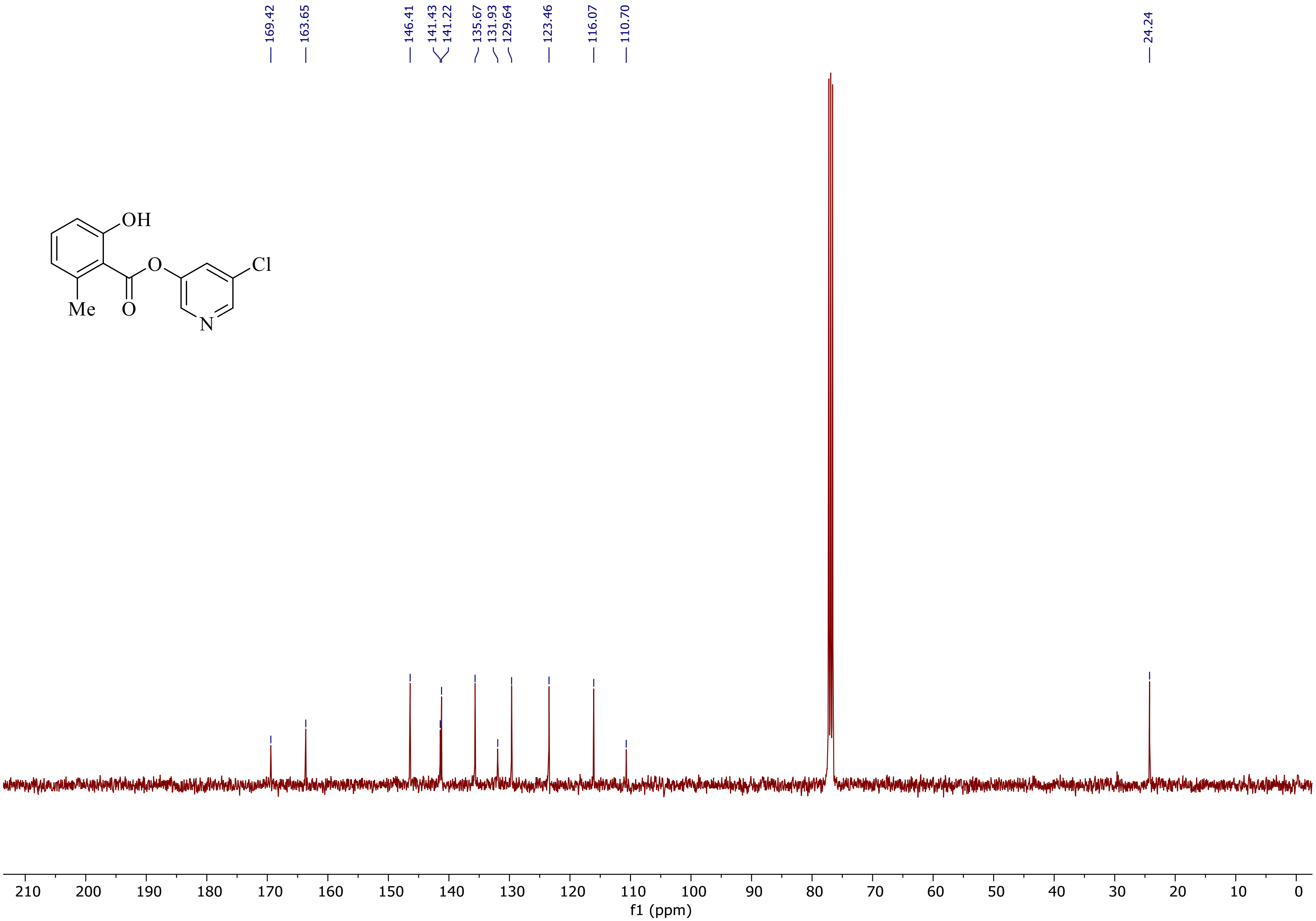

^{13}C NMR (100 MHz, $CDCl_3$) of Compound 16a

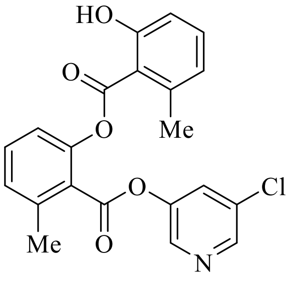

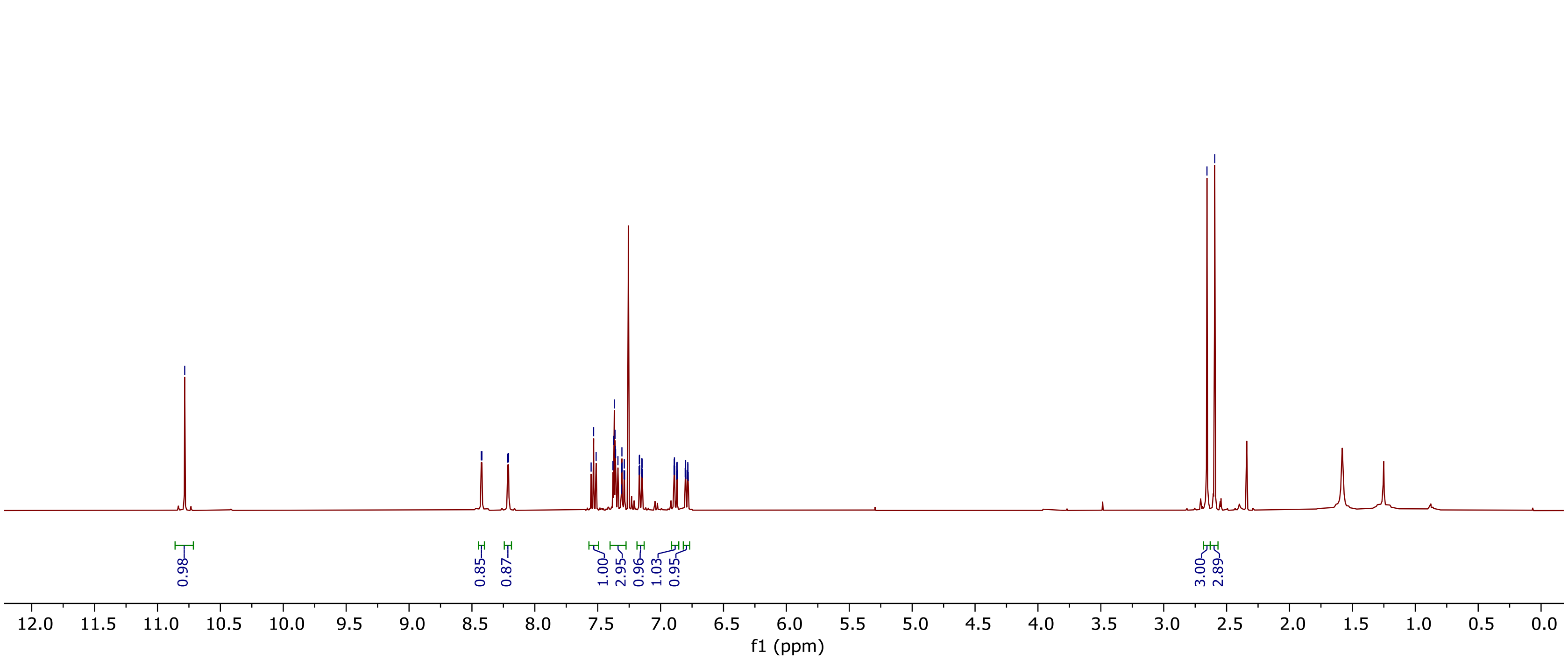

^{1}H NMR (400 MHz, $CDCl_3$) of Compound 16b

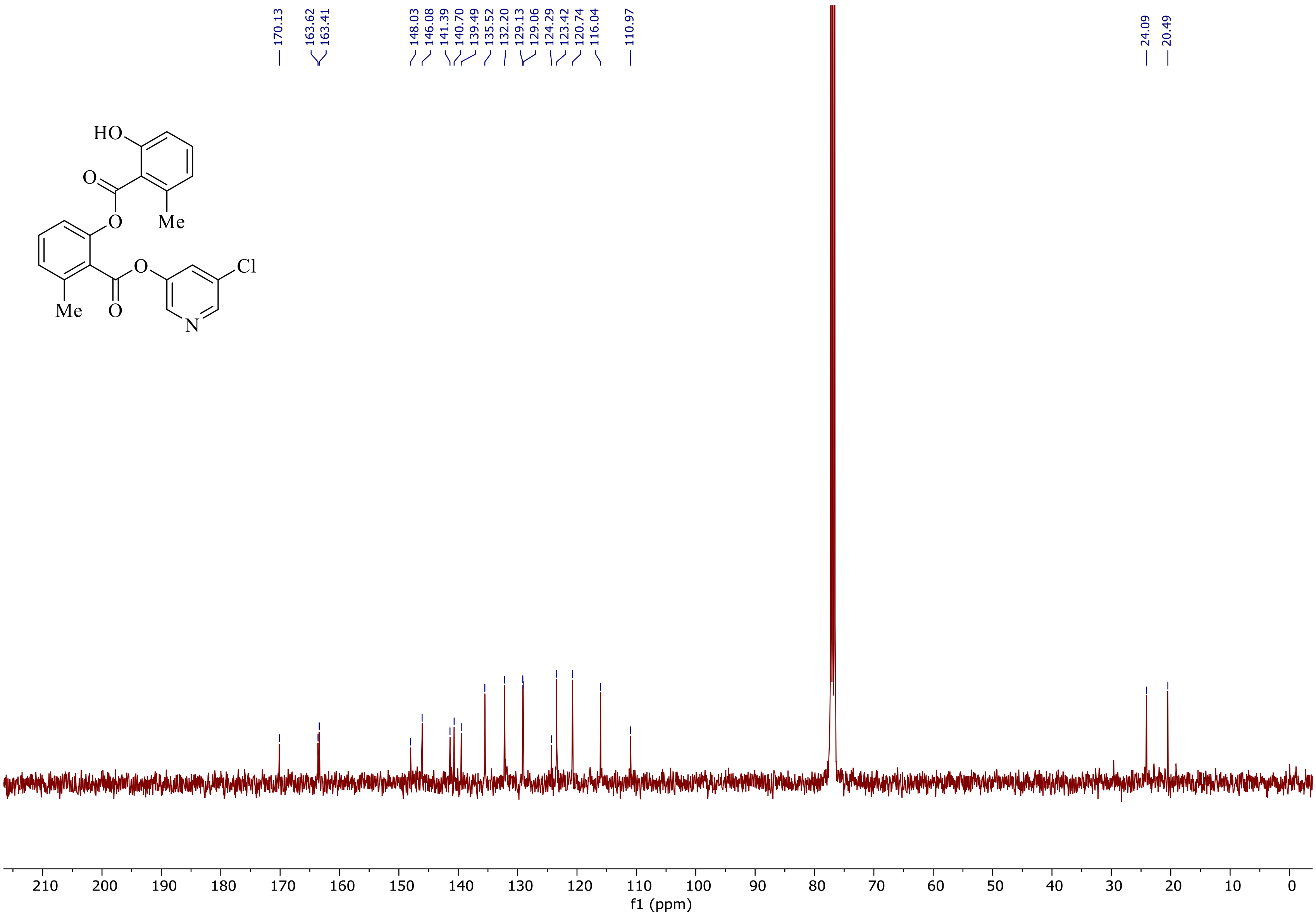

^{13}C NMR (100 MHz, $CDCl_3$) of Compound 16b

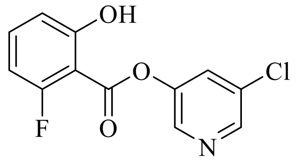

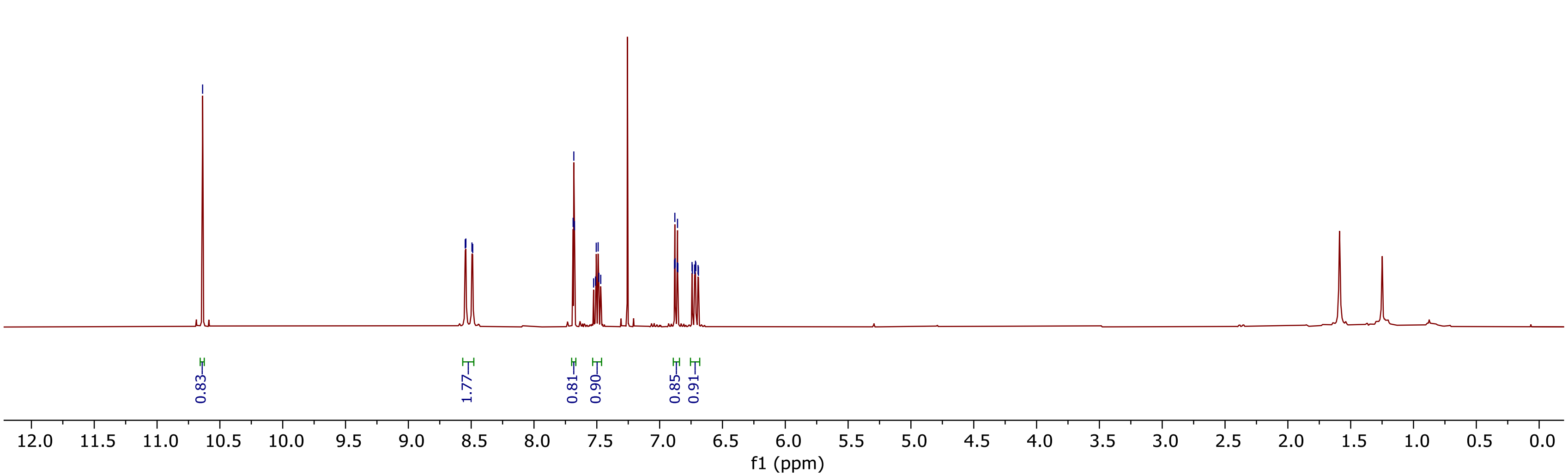
10.64
8.55
8.54
8.49
8.49
7.69
7.68
7.68
7.53
7.51
7.50
7.49
7.48
7.47
6.88
6.88
6.88
6.86
6.86
6.85
6.74
6.74
6.72
6.72
6.71
6.71
6.69
6.69
0.83
1.77
0.81
0.90
0.85
0.91
12.0 11.5 11.0 10.5 10.0 9.5 9.0 8.5 8.0 7.5 7.0 6.5 6.0 5.5 5.0 4.5 4.0 3.5 3.0 2.5 2.0 1.5 1.0 0.5 0.0
f1 (ppm)

^{1}H NMR (400 MHz, $CDCl_3$) of Compound 17a

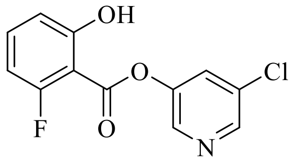

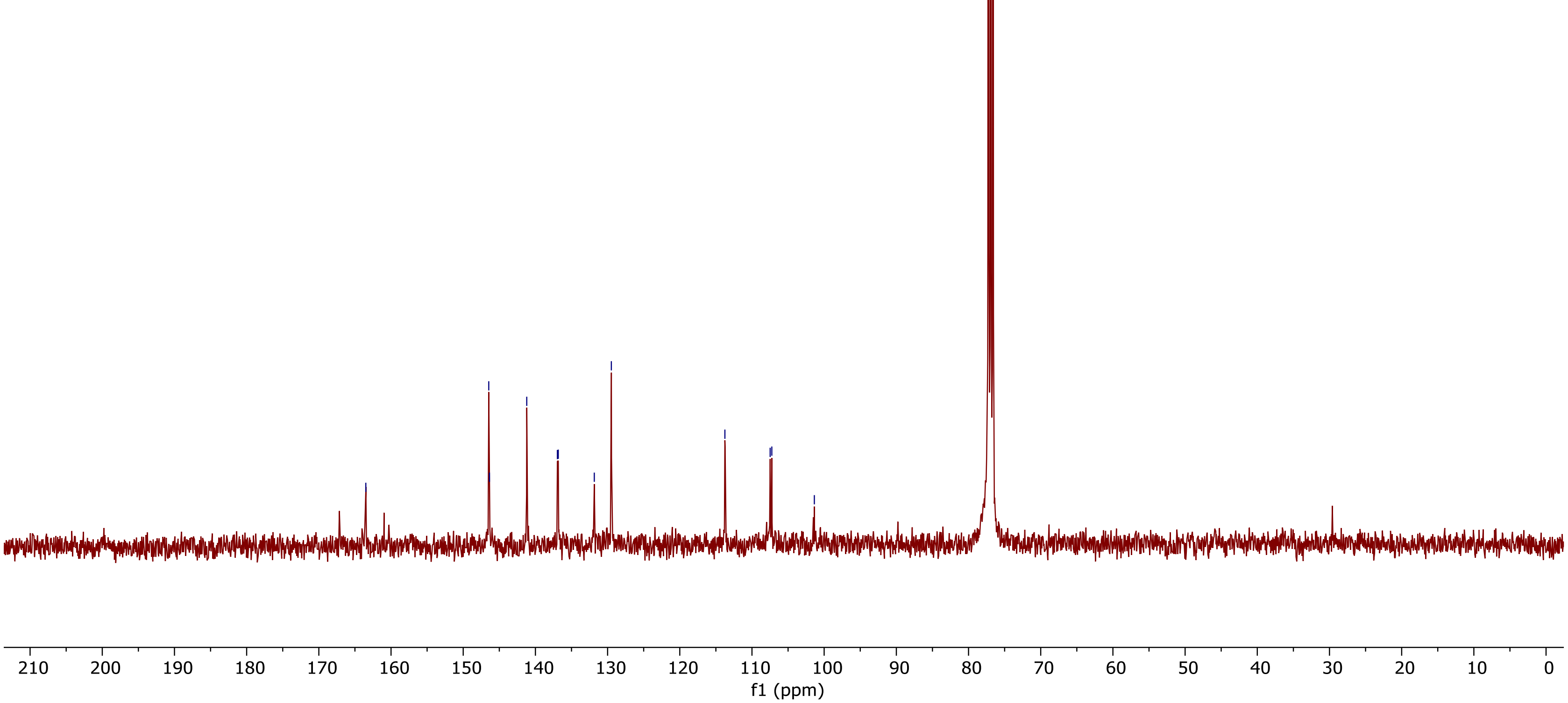

^{13}C NMR (100 MHz, CDCl$_3$) of Compound 17a

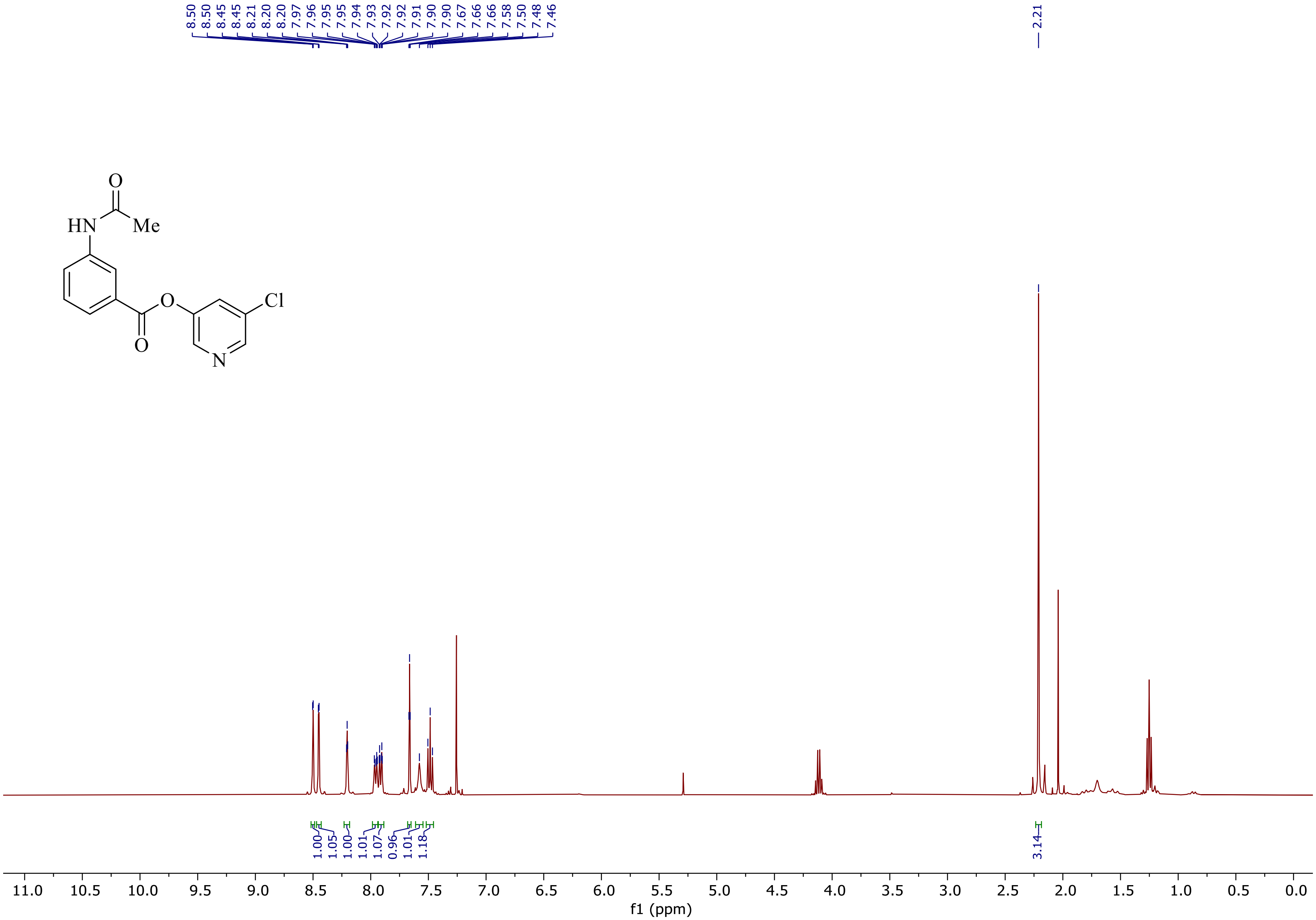

^{1}H NMR (400 MHz, $CDCl_3$) of Compound 18a

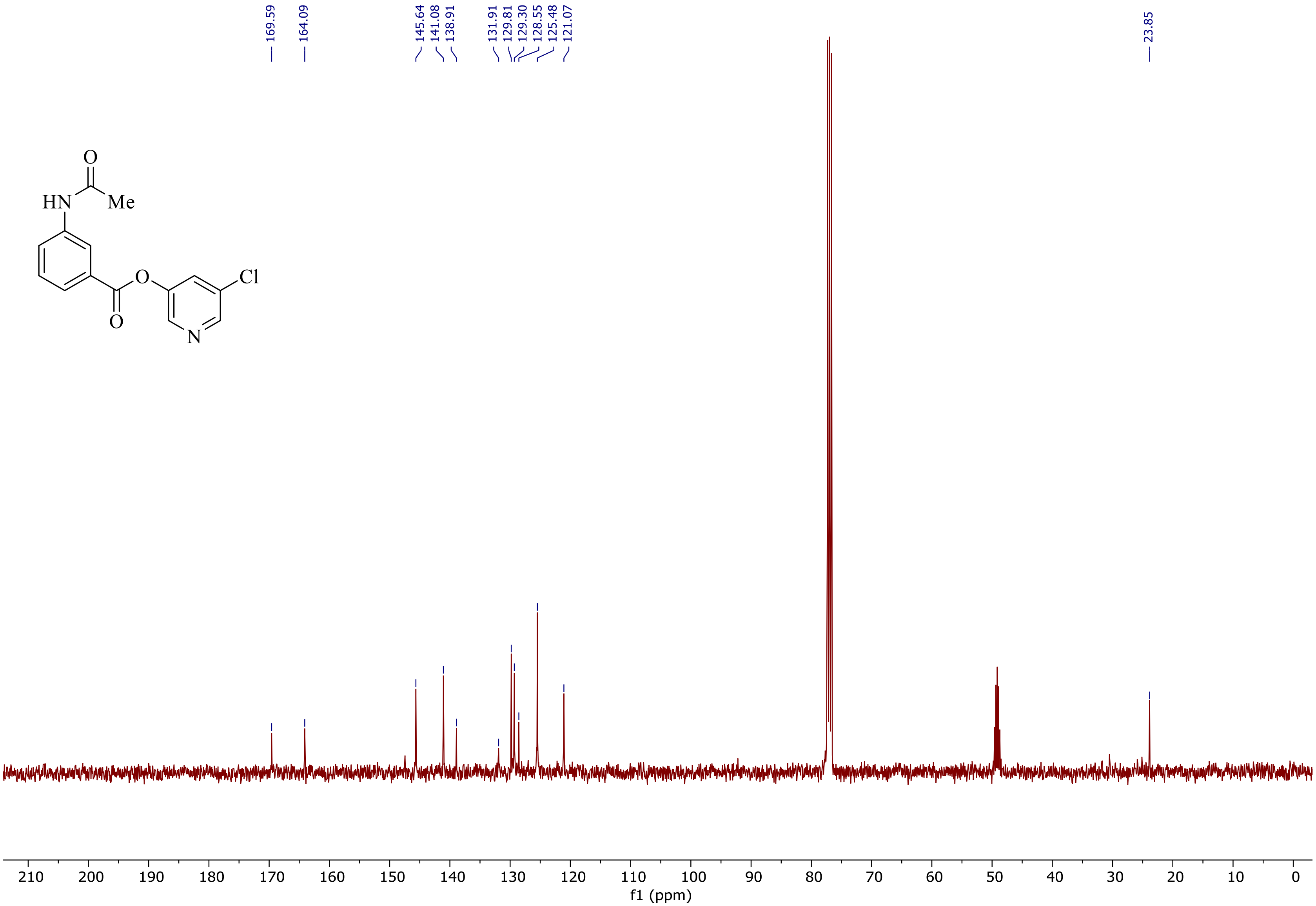

^{13}C NMR (100 MHz, $CDCl_3$) of Compound 18a

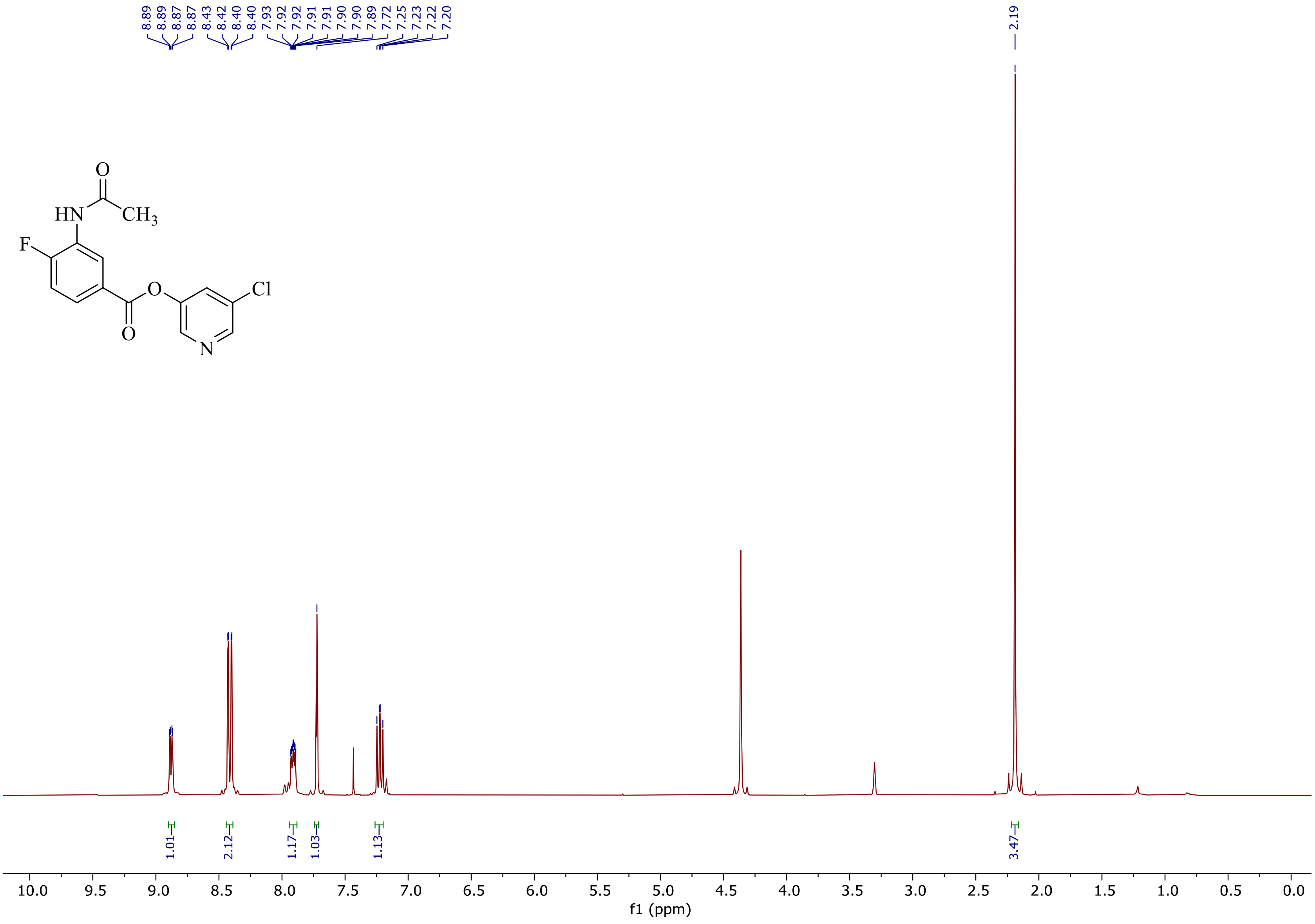

^{1}H NMR (400 MHz, MeOD) of Compound 19a

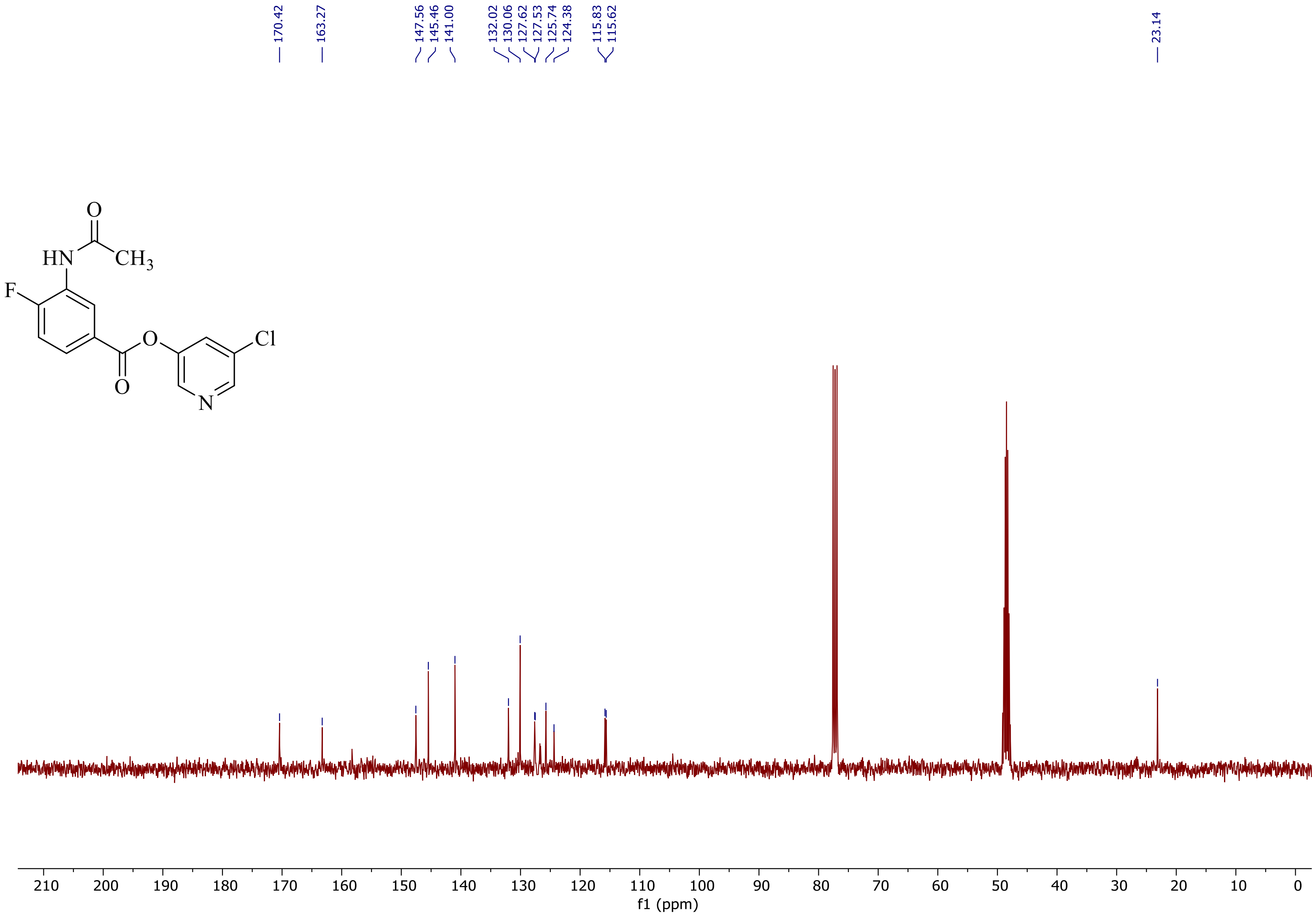

^{13}C NMR (100 MHz, MeOD) of Compound 19a
